# High-quality chromosome-level scaffolds of the plant bug *Pachypeltis micranthus* provide insights into the availability of *Mikania micrantha* control

**DOI:** 10.1186/s12864-023-09445-8

**Published:** 2023-06-20

**Authors:** Xiafei Wang, Ning Zhao, Liqiong Cai, Naiyong Liu, Jiaying Zhu, Bin Yang

**Affiliations:** 1 Key Laboratory of Forest Disaster Warning and Control of Yunnan Province, Southwest Forestry University, Kunming, China; 2 Key Laboratory for Forest Resources Conservation and Utilization in the Southwest Mountains of China, Ministry of Education, Southwest Forestry University, Kunming, China

**Keywords:** *Pachypeltis micranthus*, Chromosome-level scaffolds, *Mikania micrantha*, Genome assembly, Adaptation, Salivary gland transcriptome

## Abstract

**Background:**

The plant bug, *Pachypeltis micranthus* Mu et Liu (Hemiptera: Miridae), is an effective potential biological control agent for *Mikania micrantha* H.B.K. (Asteraceae; one of the most notorious invasive weeds worldwide). However, limited knowledge about this species hindered its practical application and research. Accordingly, sequencing the genome of this mirid bug holds great significance in controlling *M. micrantha*.

**Results:**

Here, 712.72 Mb high-quality chromosome-level scaffolds of *P. micranthus* were generated, of which 707.51 Mb (99.27%) of assembled sequences were anchored onto 15 chromosome-level scaffolds with contig N50 of 16.84 Mb. The *P. micranthus* genome had the highest GC content (42.43%) and the second highest proportion of repetitive sequences (375.82 Mb, 52.73%) than the three other mirid bugs (i.e., *Apolygus lucorum*, *Cyrtorhinus lividipennis*, and *Nesidiocoris tenuis*). Phylogenetic analysis showed that *P. micranthus* clustered with other mirid bugs and diverged from the common ancestor approximately 200 million years ago. Gene family expansion and/or contraction were analyzed, and significantly expanded gene families associated with *P. micranthus* feeding and adaptation to *M. micrantha* were manually identified. Compared with the whole body, transcriptome analysis of the salivary gland revealed that most of the upregulated genes were significantly associated with metabolism pathways and peptidase activity, particularly among cysteine peptidase, serine peptidase, and polygalacturonase; this could be one of the reasons for precisely and highly efficient feeding by the oligophagous bug *P. micranthus* on *M. micrantha*.

**Conclusion:**

Collectively, this work provides a crucial chromosome-level scaffolds resource to study the evolutionary adaptation between mirid bug and their host. It is also helpful in searching for novel environment-friendly biological strategies to control *M. micrantha*.

**Supplementary Information:**

The online version contains supplementary material available at 10.1186/s12864-023-09445-8.

## Background

**Fig. 1:**
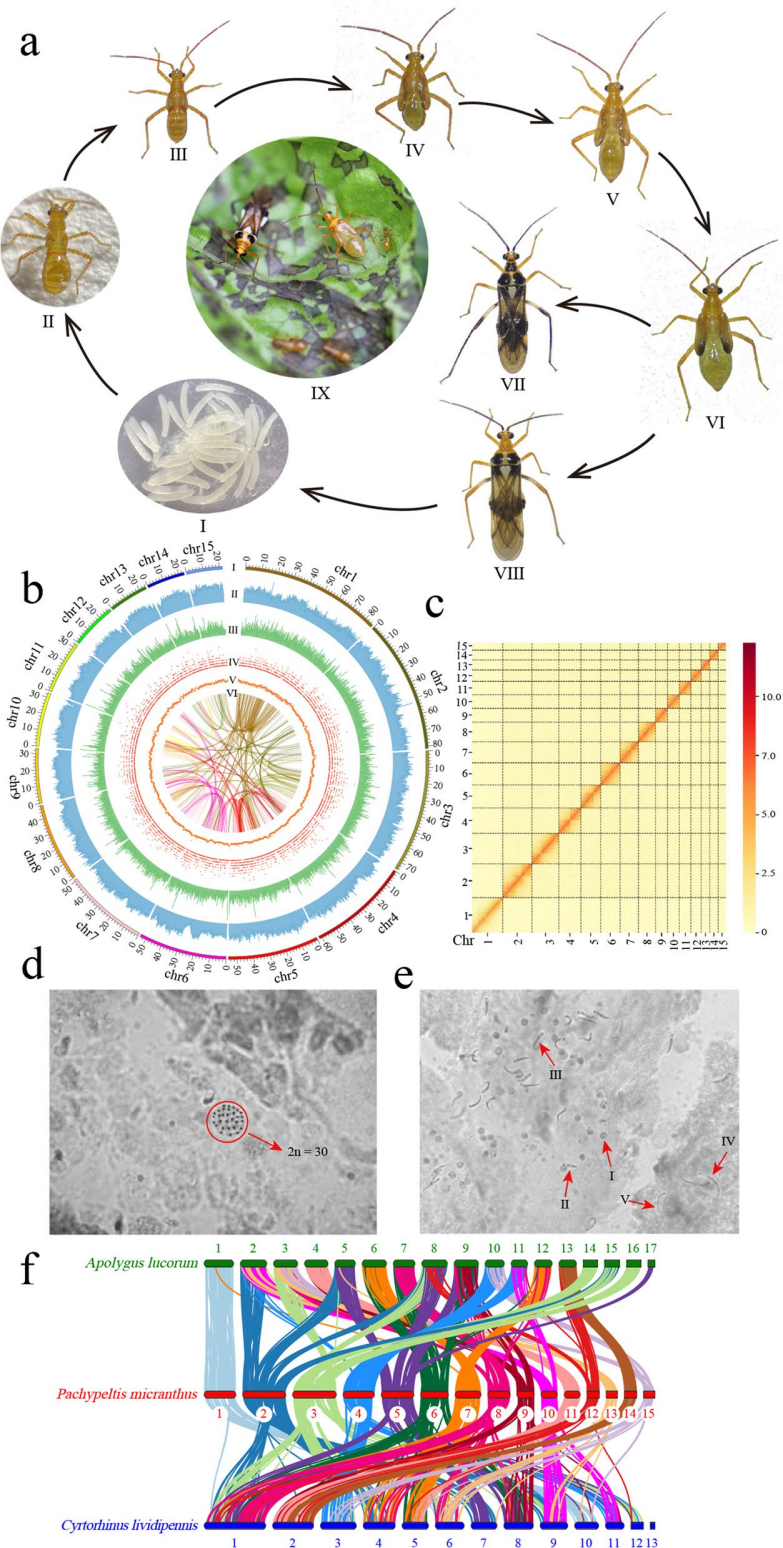
Biology and genome characteristics of *Pachypeltis micranthus*. **a** Life cycle of the plant bug *P. micranthus* (I-VIII) and the damage symptom to *Mikania micrantha* (IX). (I) Eggs, (II-VI) first to fifth instar nymph, (VII) male adult bug, (VIII) female adult bug, (IX) and damage from nymphs and adult bug feeding. **b** Genomic landscape of *P. micranthus.* From the outer to inner circles: (I) sizes of fifteen chromosome-level scaffolds, the scale bar indicates the length of the chromosome-level scaffold in Mb; (II) density of transposable elements (TEs); (III) density of tandem repeats (TRs); (IV) gene density; (V) GC density; (VI) Collinearity within the genome of *P. micranthus*. Densities are calculated in 100 Kb windows. **c** The Hi-C chromosomal interaction map for the fifteen chromosome-level scaffolds of *P. micranthus*. **d** Chromosomes of gonadal cells of *P. micranthus* in mitotic metaphase (2*n* = 30, 1000 X). **e** Different stages of sperm in *P. micranthus*. Red arrows indicate the sperms, and the numbers (I—V) next to the red arrows represent the partial process of sperm formation. **f** Chromosome-level scaffolds synteny based on CDS pairwise alignment between *P. micranthus*, *Apolygus lucorum*, and *Cyrtorhinus lividipennis*. Coloured lines indicate shared syntenic blocks

The plant bug, *Pachypeltis micranthus* Mu et Liu (Hemiptera: Miridae) (Fig. 1a_I-VIII), was first discovered in Yunnan, China, in 2008 and identified as a novel species in 2017 [1, 2]. The bug feeds gregariously on the leaves of *Mikania micrantha* H.B.K. (Asteraceae; the top 100 of the world’s worst invasive plants). Feeding by *P. micranthus* leads to leaf discolouration (Fig. 1a_IX), delays stem growth, sharply reduces the number of flowers in *M. micrantha*, and may lead to death in established plants [2, 3]. Furthermore, field and laboratory experiments have further demonstrated that, compared to other closely related plants, companion plants, economically important crops, and horticulture and landscape plants, *P. micranthus* specifically oviposits on *M. micrantha* and poses a threat to *M. micrantha*. The bug also relies on *M. micrantha* to complete its life cycle [4, 5]. Hence, *P. micranthus* can be an efficient biological agent to control *M. micrantha*.

An adequate understanding of the mechanism of *P. micranthus* feeding on *M. micrantha* is beneficial for the availability of this bug in *M. micrantha* control. *P. micranthus* is an oligophagous insect that feeds on the leaves of *M. micrantha* and small amounts of leaves from *Eupatorium odoratum*, *Ageratina adenophora* (Sprengel) R. King and H. Robinson, and *Gynura crepidioides* Benth [4]. Insects can recognize their plant hosts based on chemical signals emitted by the plants with the chemosensory systems [i.e., odorant-binding proteins (OBPs), chemosensory proteins (CSPs), and odorant receptors (ORs)] [6, 7]. The chemosensory system plays a crucial role not only in the processes of locating food but also shelter, mates, and oviposition [8, 9]. Therefore, many studies have reported extensive chemosensory genes in other mirid bugs (*Apolygus lucorum*, *Cyrtorhinus lividipennis*, *Lygus lineolaris*, and *Lygus hesperus*) [10, 11]. Myrcene was already confirmed as one of the most abundant volatiles in *M. micrantha* and showed a potent attractive effect on this bug [12]. So far, however, only nine OBPs, three CSPs, and one OR gene have been reported in *P. micranthus* [13], which is insufficient to study the attraction mechanism of *M. micrantha* to this bug.

In most insects, salivary glands are important labial glands that secrete saliva, an essential chemical substance with biological activities and complex composition, including many digestive enzymes (e.g., proteinases, phospholipase, esterase, serine proteases, trehalase) [14–16]. Like other mirid bugs, *P. micranthus* feed by inserting its stylet into plant tissues and injecting enzyme-containing saliva (digestive enzymes); the injected saliva is responsible for stylet lubrication and preliminary digestion of plant tissues [14, 17, 18]. The salivary enzymes remaining in the feeding site cause continuous tissue damage for an extended period, leading to a decrease in the growth rate and loss of flowers [19–21]. The primary damage caused by mirid bugs during feeding is due to saliva rather than mechanical damage caused by stylet [20]. Moreover, the component in saliva also has a detoxification effect and acts as an effector to induce or inhibit plant defence responses [22]. Therefore, mirid bugs feeding can trigger severe damage to plants, such as leaf discolouration, necrosis of the feeding site, organ abscission, flower bud abortion, and even the death of the entire plant [23, 24]. Based on these symptoms, *P. micranthus* can control *M. micrantha*, but harmful agricultural mirid bugs can cause crop yield reduction [2, 17]. In the past, salivary gland transcriptome analysis has mainly focused on blood feeding [18]. For mirid bugs, only the salivary gland transcriptomes of *Lygus lineolaris* were reported [18, 21], providing valuable information for omnivorous mirid bugs. However, the salivary gland transcriptome of *P. micranthus* is needed, and generating this data will increase our knowledge of how the oligophagous mirid bugs can adapt to the specific host.

Plants can produce many specialized chemical substances that resist the herbivores’ challenges. Herbivory can seriously reduce the survival rate and fecundity of local plants. In crops, invading herbivorous insects will lead to severe yield loss [25, 26]. Herbivore-induced plant defence is divided into direct defences, such as toxins or anti-digestive proteins, and indirect defences, such as the plant volatiles that attract the natural enemies of herbivores [27]. Continued exposure to toxic or anti-digestive compounds is a defence option for herbivores to adapt to host plants, often making better-defended plants the targets of herbivores. The process of this co-evolution leads to the host plant specialization of insects. Thus, most herbivores feed on only a few host plants [26]. Insects rely primarily on four detoxification enzyme families, including cytochrome P450s (P450s), glutathione S-transferases (GSTs), carboxylesterases (CCEs), and ATP-binding cassette transporters (ABCs), to metabolize the toxic substances from the food and environment [28, 29]. Liu et al. reported the *A. lucorum* genome and many detoxification enzymes of glutathione GSTs and P450s, explaining a better detoxification system of toxins and adaptation to the environment in *A. lucorum* [11]. There are numerous bioactive secondary metabolites in *M. micrantha* tissues [30]. Notably, although the extracts from *M. micrantha* show insect avoidance, toxicity, and antibacterial activity [31–33], the feeding and oviposition of *P. micranthus* still rely on *M. micrantha* [34]. This finding may suggest that *P. micranthus* has evolved a potent detoxification ability to defend against xenobiotics from *M. micranthus*. The information on the *P. micranthus* genome will contribute to figuring out how this bug can resist its hosts’ defence.

Invasive species have received worldwide attention, and they have caused severe economic loss and negative environmental impacts [35]. There is already a multitude of methods to control invasive species, mainly including mechanical [36], chemical [37], and traditional biological control (introduce the natural enemies of invaders) [38]. However, these methods are inefficient in controlling invasive species and cause diverse environmental impacts on ecosystems [39]. The interactions between native or naturalized non-invasive and invasive species can prevent invasions [40]. Furthermore, the co-evolution of native species and other local species matched with local phenology and will not limit non-target species. Therefore, using native species to control invasions could reduce the impact on non-target species and is expected to replace traditional biological control [40, 41]. Using native insects to control invasive plants is effective and cost-efficient [42]. Native insects can persist and reproduce naturally without excessive human intervention. The biological control agents and target species continuously adapt, making the development of resistance nearly impossible. This control method can also reduce chemical pesticides' short-term or long-term impacts on human health and the environment [43, 44]. In general, *P. micranthus*, like other invertebrate biological control agents (both exotic and native), pose a shallow risk to human and animals, and there are virtually no reports of adverse effects in the literature [45]. The side effects of other invertebrate biological control agents are limited to occasional bites, stings, and allergic reactions [46], which have not been reported in *P. micranthus*. *P. micranthus*, a specialized and effective native enemy of *M. micrantha* [1], belongs to the family Miridae, a species-rich family of plant bugs in Hemiptera, and contains an estimated 11,020 recorded species. Due to the broad range of food preferences and behaviours, mirid bugs can be divided into phytophagy, carnivory, and omnivory [17, 47]. Most mirid bugs are pests that infest primary food and fibre crops, but a few species are natural enemies [17, 47, 48]. However, apart from *P. micranthus*, only three genome assemblies of Miridae have been published in the NCBI database (based on the NCBI genome database as of March 23, 2023), two of which are harmful polyphagous herbivores (*A. lucorum*, *Nesidiocoris tenuis*) and another bug (*C. lividipennis*) is a nature enemy of harmful herbivores [11, 49, 50]. The resources of the natural enemy genome are precious in the research of developing environmental-friendly and efficient new biological control strategies.

To provide the essential resources for researching new biological control strategies and understanding how *P. micranthus* adapts to the malignant invasive weed *M. micrantha*. High-quality chromosome-level scaffolds of *P. micranthus* were generated in this study using various sequencing methods. Some significantly expanded gene families associated with feeding and host adaptation had been confirmed through gene expansion and contraction analysis. Then, three significant classes (i.e., chemoreceptor, digestion, and detoxification) of gene families that may be related to *P. micranthus* adaptation to *M. micrantha* were manually identified. In addition, the salivary gland transcriptome of *P. micranthus* was analyzed to elucidate the specific adaptation of this bug to *M. micrantha* and the mechanism of highly efficient feeding by this mirid bug on *M. micrantha*.

## Results and discussion

### Genome sequencing and de novo assembly

The *Pachypeltis micranthus* genome was sequenced using Nanopore GridION X5, PacBio, and BGI MGISEQ-2000 platforms. After filtering, 75.52 Gb Nanopore pass reads, 12.36 Gb PacBio long reads, and 113.67 Gb Illumina short reads were generated, with 98.87X, 30.20X, and 154.80X genome coverages (Table 1). The Illumina short reads were used to estimate the size and heterozygosity of the *P. micranthus* genome with k-mer analysis. The distribution of 17-mers frequency indicated that the genome of *P. micranthus* was 708.32 Mb with 0.9% of heterozygosity (Additional file 1: Fig. S1 and Additional file 2: Table S1). The heterozygosity was similar to *A. lucorum* (1%) [11] and lower than *C. lividipennis* (1.7%) [50]. This difference could be related to the polymorphism of the sequenced natural population.

**Table 1:** Summary of genome sequencing of *Pachypeltis micranthus* in this study

Library	Platform	Sample	Insert size (bp)	Raw data (Gb)	Clean data (Gb)
Nanopore	Nanopore	Male adult	20,000	NA	75.52
PacBio	PacBio	Male adult	20,000	21.50	12.36
Illumina	MGISEQ-2000	Male adult	350	131.52	113.67
Hi-C	Illumina Novaseq	Male adult	300–600	112.00	110.96
RNA-seq	Illumina HiSeq	Male adult	150	7.80	7.71
RNA-seq	Illumina HiSeq	Salivary glands	150	8.77	8.67

An initial de novo assembly was done using the NextGraph module, and an assembly of 710.81 Mb with a contig N50 of 16.82 Mb was obtained. The initial assembly contigs were polished correction for seven rounds with NextPolish v1.3.0 [51], using PacBio long reads and Illumina short reads. After polishing the initial assembly, a reference *P. micranthus* genome of 712.72 Mb with contig N50 of 16.84 Mb was obtained (Table 2, Additional file 2: Table S2), which is close to the estimate by k-mer analysis. The line graph of the length of the contigs showed that the assembled genome has a good continuity (Additional file 1: Fig. S2). The genome of *P. micranthus* is about 0.5-fold smaller than that of *A. lucorum* and about twofold larger than that of *C. lividipennis* and *N. tenuis* (Table 2). The *P. micranthus* genome revealed a relatively high GC content (42.43%) compared to other mirid bugs (Table 2). Combined with the previous study, this result may illustrate a high GC content in Miridae genomes [50].

**Table 2:** Assembly statistics for four mirid bugs

Species	*Pachypeltis micranthus*	*Apolygus lucorum*	*Cyrtorhinus lividipennis*	*Nesidiocoris tenuis*
**Sequencing info**				
Sequencing technology	Nanopore + PacBio + Illumina + Hi-C	Illumina + PacBio + Hi-C	Illumina + PacBio + Hi-C	Illumina
Genome coverage	98.87X + 30.20X + 154.80X + 152.32X	100X (PacBio)	139X + 129X + 316X	NA
**Assembly info**				
Assembly level	Chromosomes	Chromosomes	Chromosomes	Scaffolds
Genome size (Mb)	712.72	1023	345.95	355.12
Number of contigs	91	3818	3784	51,853
Number of scaffolds	71	191	1615	36,513
**Genome assembly quality**				
Contig N50 (bp)	16,840,000	785,000	169,640	13,374

The genome assembly completeness was assessed using Benchmarking Universal Single-Copy Orthologs (BUSCO) v4.0.5 [52] according to the Orthologs database insecta_odb10, 1310 out of 1367 (95.83%) highly conserved insect orthologs were found in the genome (Additional file 2: Table S3). The BUSCO score of *P. micranthus* indicated the second highest among four mirid bugs in Miridae, following *A. lucorum* (Table 2). Furthermore, a total of 248 core genes (91.13%) were predicted using Core Eukaryotic Genes Mapping Approach (CEGMA) v2 [53] (Additional file 2: Table S4). The Illumina reads were remapped to the genome using BWA (Burrows-Wheeler Aligner) v0.7.12 [54] and mem algorithm with defaults parameters, and 99.86% of the reads were mapped to the assembly with a 98.24% mapping rate and 154.80X coverage depth (Additional file 2: Table S5). The single-base accuracy of 99.998453% (depth ≥ 5) in the assembled genome (Additional file 2: Table S6). For GC-depth analysis, the genome distributions of GC content focus on 30–40% with 70-90X coverage depth (Additional file 1: Fig. S3). Finally, 98.18% of genome sequences were aligned with metazoa in the nucleotide sequence database (NT). These results indicated that the assembled *P. micranthus* genome was complete and had a low error rate.

### Chromosome-level scaffolds assembly

A total of 112 Gb of raw data from the Hi-C library was generated. Moreover, 110.96 Gb of clean data were retained after filtering by removing adapter sequences and low-quality reads (Table 1). The clean data was mapped on the assembled genome, and 35.84 Gb unique mapped paired-end reads were obtained to assess valid data (Additional file 2: Table S7). Based on the assessment result, 12.63 Gb (17.72X) of high-quality validated paired-end reads were used to assemble contigs at the chromosome-level scaffolds (Additional file 2: Table S8). After clustering, ordering, and orienting, the assembled 71 contigs were anchored to 15 chromosome-level scaffolds with a length range of 21.93–85.62 Mb, and the final chromosome-level scaffolds size and N50 were 707.51 Mb and 48.15 Mb and contained 99.27% of genome sequences (Additional file 2: Table S9 and Fig. 1b_I). Hi-C heatmap showed strong interactions between adjacent sequences (Fig. 1c). For further confirmation of the results, the chromosome-level scaffolds of male bugs were observed using microscopy, and the chromosome number of *P. micranthus* was 30 (2n), which was accordant with the assembly result (Fig. 1d). Collinearity analysis showed high collinearity between those chromosome-level scaffolds, which revealed a recent gene replication and transposition (Fig. 1b_VI). Sperm morphology may be essential in determining fertilization's success and help settle taxonomic problems [55, 56]. In this study, the different stages of *P. micranthus* sperm were further observed using microscopy (Fig. 1e), which may contribute to the taxonomic research of mirid bugs.

Synteny analysis of the *P. micranthus* chromosome-level scaffolds with *A. lucorum* and *C. lividipennis* were performed using MCScanX (Python version) [57] based on a coding sequence (CDS) pair-wise synteny search. This study defined a syntenic block as containing at least three orthologous genes. *P. micranthus* showed a high level of collinearity with the two other mirid bugs with chromosome-level scaffolds, which indirectly supported the high quality of the *P. micranthus* genome assembly (Fig. 1f). 802 syntenic blocks between *A. lucorum* and *P. micranthus* were found, and the gene numbers in these blocks ranged from 4 to 24, with an average of 6.59. Comparing *P. micranthus* and *C. lividipennis*, 496 syntenic blocks were found with 4–22 genes and a mean of 5.77. Moreover, 527 syntenic blocks between *A. lucorum* and *C. lividipennis* were found with a range of 4–26 with an average of 5.88 (Additional file 1: Fig. S4). Although previous and our studies demonstrated that the evolutionary relationship of *A. lucorum* and *C. lividipennis* was closer than that of *A. lucorum* and *P. micranthus* [50], *A. lucorum* showed higher collinearity with *P. micranthus* than *C. lividipennis*. In addition, many chromosome-level scaffolds fused and corresponded to other chromosome-level scaffolds among the three species (Fig. 1f, Additional file 1: Fig. S4). These results may be caused by differences in gene density, gene transpositions, tandem duplication, genome rearrangements, and chromosome evolution [57, 58].

### Genome annotation

375.82 Mb sequences were identified as repeat sequences, constituting 52.73% of the *P. micranthus* genome (Additional file 2: Table S10). The transposable elements (TEs) in *P. micranthus* were mostly long interspersed nuclear elements (LINE, 18.16% of the genome) and DNA transposons (19.19% of the genome). The most TE and tandem repeats (TRs) were distributed on chromosome-level scaffold 13 (291.232 per 100 Kb windows, Fig. 1b_II) and chromosome-level scaffold 1 (4.81 per 100 Kb windows, Fig. 1b_III), respectively.

A total of 11,746 protein-coding genes were predicted in the genome of *P. micranthus* using three methods (homolog searching, transcriptome sequencing, and de novo prediction). With average gene length, average CDS length, average exons number per gene, average exon length, and average intron length were 32,170.81 bp, 1516.18 bp, 7.82, 193.96 bp, and 4496.87 bp, respectively (Additional file 2: Table S11), which is similar to those of other Hemiptera species (Additional file 2: Table S12 and Additional file 1: Fig. S5). In addition, approximately 92.25% of predicted protein-coding genes (10,836 genes) could be functionally annotated (Additional file 2: Table S13). BUSCO analysis showed that 96.20% (single-copy genes: 95.10%, duplicated genes: 1.10%) of protein-coding genes were predicted as complete, 0.73% of those genes were fragmented, and 3.07% of those genes were missing (Additional file 2: Table S14), further emphasizing the accuracy and completeness of gene predictions. The gaps with shorter scaffolds or contigs would increase the pseudogenes or false positive annotations [59]. Although the *P. micranthus* genome is about twofold larger than that of *C. lividipennis* and *N. tenuis* mentioned above, the number of protein-coding genes in the *P. micranthus* genome is less than those of three other mirid bugs (Table 2). This difference may be related to its high content of repetitive sequences.

Different types of non-coding RNAs (ncRNAs) were also annotated, including 263 ribosomal RNAs (rRNAs), 63 small nuclear RNAs (snRNAs), 39 microRNAs (miRNAs), 40 regulatory RNAs, and 2501 transfer RNAs (tRNAs) (Additional file 2: Table S15).

### Phylogenetic analysis

**Fig. 2:**
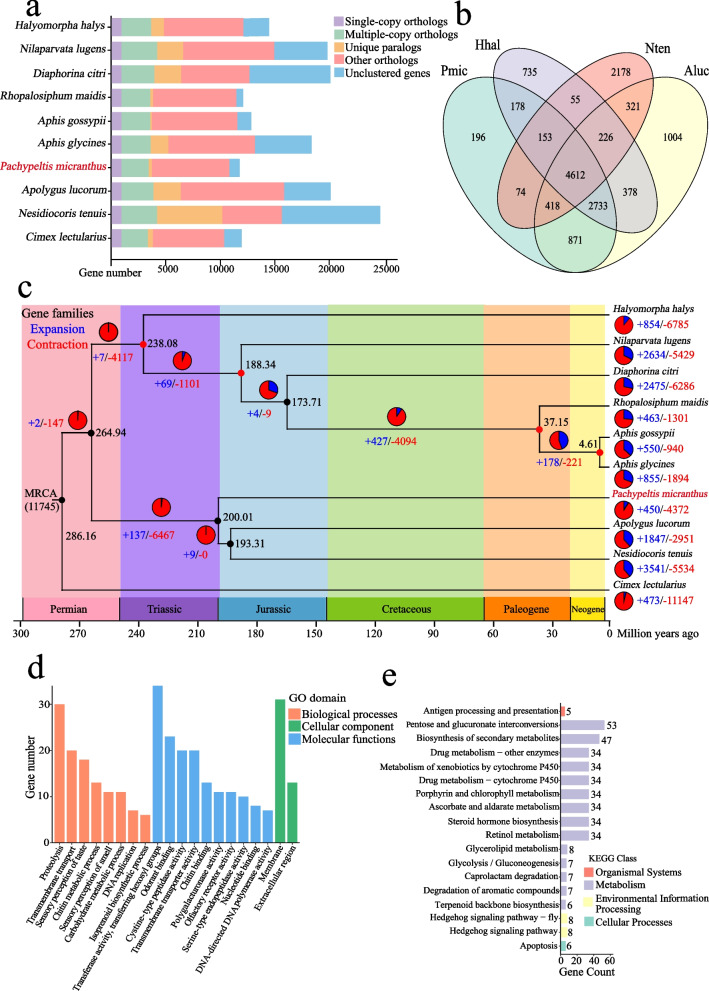
Comparative genomic analysis among *Pachypeltis micranthus* and nine other species. **a** Gene ortholog of *P. micranthus* with nine other species. Bars showing gene counts are subdivided to represent classes of orthology. **b** Venn diagram showing the distribution of orthologous clusters between *P. micranthus* (Pmic), *Halyomorpha halys* (Hhal), *Nesidiocoris tenuis* (Nten), and *Apolygus lucorum* (Aluc) (only these three Hemipteran with close evolutionary relationship to *P. micranthus* are shown for clarity). Numbers indicate gene families identified among all selected species. **c** The phylogenetic tree was built based on 896 single-copy orthologous genes of ten species. Numbers below each species' name indicate the number of expanded (blue) and contracted (red) gene families in each insect. The number next to each branch represents the number of expanded (blue) and contracted (red) gene families in each clade. The numbers next to each node indicate the divergence time. The nodes with known fossil time are labelled red and used for time calibration. **d** Gene Ontology (GO) enrichment analysis (*p* < 0.05) of the significantly expanded gene family in the *P. micranthus* genome. **e** KEGG pathway enrichment analysis (*p* < 0.05) of the significantly expanded gene family in the *P. micranthus* genome

OrthoMCL v2.0.9 [60] was used for gene family clustering analysis among *P. micranthus* and the nine other Hemiptera species, including *N. tenuis*, *A. lucorum*, *Aphis gossypii*, *Nilaparvata lugens*, *Diaphorina citri*, *Halyomorpha halys*, *Aphis glycines*, *Rhopalosiphum maidis*, and *Cimex lectularius*. Furthermore, 896 single-copy orthologous genes and 2487 multiple-copy genes were identified in *P. micranthus* (Fig. 2a and Additional file 2: Table S16). The coding sequences of the single-copy genes were used to construct a phylogenetic tree and estimate divergence time. The phylogenetic relationships indicated that *P. micranthus* was a basal hemipteran species and the ancestor of *N. tenuis* and *A. lucorum* split with *P. micranthus* approximately 200 million years ago (Mya) (Additional file 1: Fig. S6).

In the gene family analysis, unique and unclustered genes were considered species-specific. Based on the OrthoMCL results above, a total of 1223 species-specific genes were identified in the *P. micranthus* genome (Additional file 2: Table S17), which were significantly enriched in Gene Ontology (GO; Gene Ontology Consortium, geneontology.org) [61] categories, including proteolysis (GO:0006508), structural constituent of cuticle (GO:0042302) and serine-type endopeptidase activity (GO:0004252) (Additional file 1: Fig. S7). The homologous gene groups were then compared between *P. micranthus* and the other three hemipteran species: 4612 gene families were shared by *P. micranthus*, *H. halys*, *N. tenuis,* and *A. lucorum*, and 178 gene families were shared by *P. micranthus* and *H. halys* while not the other two hemipterans; and only 74 gene families were shared by *P. micranthus* and *N. tenuis* while not with the other two; 871 genes families were shared between *P. micranthus* and *A. lucorum* but not with the other two (Fig. 2b). Furthermore, *N. tenuis* shared the most gene families with *A. lucorum* (5577) than *P. micranthus* (5272) and *H. halys* (5046), which was consistent with the evolutionary relationships (Fig. 2b).

### Evolution of gene families

Gene family expansion and contraction have been suggested as essential and fundamental adaptation mechanisms [62]. To reveal important gene family changes related to adaptation, gene family expansion and contraction in the *P. micranthus* genome were analyzed using CAFE v4.2.1 [63] by comparing with the genomes of *A. lucorum*, *N. tenuis*, *A. gossypii*, *N. lugens*, *D. citri*, and *H. halys, A. glycines*, *R. maidis* and *C. lectularius*.

Gene family expansion and contraction manifest as changes in the number of genes within gene families [64]. Copy number variations and accumulation of mutations expanded the size of gene families, whereas gene reductions decreased the size of gene families [65]. In the genome of *P. micranthus*, 450 and 4372 gene families were expanded and contracted, respectively (Fig. 2c). This finding suggested that many gene families in the *P. micranthus* genome were lost rather than gained during adaptive evolution. Additionally, significant variations were observed in the quantification of gene family expansions and contractions on each branch, with contractions outnumbering expansions by a large margin (Fig. 2c). The expansion and contraction of specific gene families are often associated with adaptive differences among closely related species [66]. Additionally, the gain and loss of genes are also associated with functional changes and contribute to variations in morphology, physiology, and metabolism among species [67, 68]. Hemipteran insects exhibit diverse species and possess various food sources (ranging from plants, fungi, small arthropods, and vertebrate blood) along with strong adaptability [17, 47, 69]. The prevalence of gene family contractions over expansions suggests that gene loss may play a significant role in the adaptive evolution of Hemipteran insects, and gene loss is likely a result of vertical descent, where ancestors directly transmit genes to their descendants.

The significantly expanded and contracted gene families (*p* < 0.05) in the genome of *P. micranthus* were selected and subjected to GO and Kyoto Encyclopedia of Genes and Genomes (KEGG; www.genome.jp/kegg/) [70] functional enrichment analysis. The GO analysis revealed that the expansion genes were significantly enriched in various GO terms, such as transferase activity, membrane, proteolysis, odorant binding, cysteine-type peptidase activity as well as sensory perception of taste (Fig. 2d). KEGG enrichment analysis showed that most of the expanded genes were significantly enriched in carbohydrate metabolism, biosynthesis of secondary metabolites, metabolism of cofactors and vitamins, xenobiotics biodegradation and metabolism, lipid metabolism, signal transduction, cell growth and death, metabolism of terpenoids and polyketides and immune system (Fig. 2e). Significant expansion or contraction of gene families is commonly associated with the adaptive evolution of species [71, 72]. *P. micranthus* absorbs nutrients required to support its development and growth from *M. micranthus* leaves, and its oviposition also relays on *M. micranthus* stems. Thus, several significantly expanded gene families associated with chemoreceptor, digestion, and detoxification were considered necessary for adaptive evolution. These include the chemoreceptor annotations, such as odorant binding and sensory perception of taste; the digestion annotations, such as proteolysis and polygalacturonase activity; the detoxification annotations, such as drug metabolism-cytochrome P450 and metabolism of xenobiotics by cytochrome P450 (Additional file 2: Tables S18 and S19). However, GO and KEGG analysis of the contracted gene families revealed that only one gene was significantly enriched in DNA-templated transcription, nucleus, and basal transcription factors (Additional file 2: Tables S20 and S21).

In addition, 11 positively selected genes were identified in *P. micranthus*, and then KEGG and GO analyses were done (Additional file 2: Tables S22 and S23). These genes involved some terms, such as lipid metabolism, genetic information processing, and signal transduction.

### Gene family analysis potentially associated with *P. micranthus* host adaptation

Insect feeding behaviour is a complex process associated with initial activation, orientation, identification, and feeding [73]. As stated, the chemosensory system is crucial in locating food, mates, and oviposition [8, 9]. *P. micranthus* is an oligophagous insect, and myrcene has a robust and attractive effect on this bug [12]. It has been shown that olfactory cues could explain the physiological mechanism underlying host recognition [7, 74]. *P. micranthus* feed by inserting piercing-sucking mouthpart into *M. micrantha* leaves, injecting slavery enzymes, and then aspirating liquefied materials. Therefore, this plant bug is a typical extraoral digestion, piercing-sucking, and “lacerate and flush feeding” insect [17]. The liquefied materials are further digested and absorbed in the gut. As previously stated, the digestive enzymes remaining in plants cause continuous tissue damage for an extended period, leading to a decrease in the growth rate and loss of flowers [19–21]. The digestive enzyme is an essential factor in the adaption of *P. micranthus* to *M. micrantha* and also provides a new strategy to control *M. micrantha*. The adaption of insects to the plants they feed on partially depends on detoxification genes [75]. Thus, the detoxification genes can further support *P. micranthus* feeding and adaptation to *M. micrantha*.

**Fig. 3:**
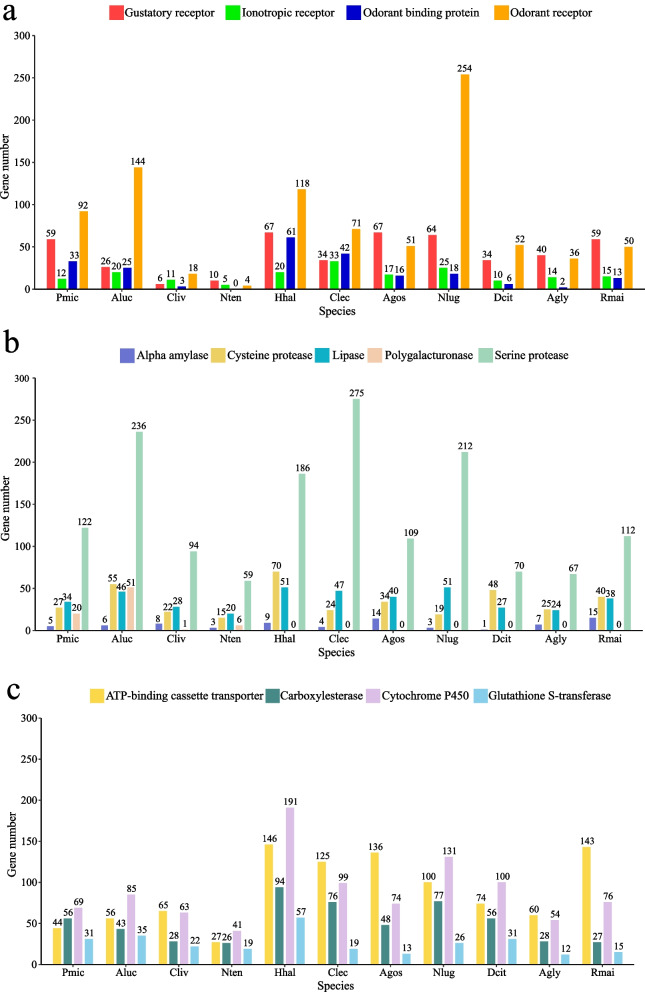
Comparison of candidate gene families associated with host adaptation among 11 Hemiptera insects [*Pachypeltis micranthus* (Pmic), *Apolygus lucorum* (Aluc), *Cyrtorhinus lividipennis* (Cliv), *Nesidiocoris tenuis* (Nten), *Halyomorpha halys* (Hhal), *Cimex lectularius* (Clec), *Aphis gossypii* (Agos), *Nilaparvata lugens* (Nlug), *Diaphorina citri* (Dcit), *Aphis glycines* (Agly), and *Rhopalosiphum maidis* (Rmai)]. **a** Counts of four chemoreceptor gene families [(Gustatory receptor (GR), Ionotropic receptor (IR), Odorant binding protein (OBP), and Odorant receptor (OR)]. **b** Counts of five digestion gene families [(Alpha-amylase, Cysteine protease (CP), Lipase, Polygalacturonase (PG), and Serine protease (SP)]. **c** Counts of four detoxification gene families [Glutathione S-transferase (GST), Cytochrome P450 (P450), Carboxylesterase (CCE), and ATP binding cassette transporter (ABC)]

In the *P. micranthus* genome, 59 gustatory receptors (GRs), 12 ionotropic receptors (IRs), 40 OBPs, and 92 ORs were manually identified (Fig. 3a), which were closely related to the encoding of significantly expanded gene families. This relevance may also indicate the specific recognition and adaptation mechanism of *M. micrantha*. GRs, ORs, and IRs are thought to help detect odours and function as chemosensory receptor multi-gene families in insects [9]. The three chemosensory receptor gene families are mainly expressed in insect chemosensory sensilla that harbour olfactory sensory neurons (OSNs) [76]. GRs are expressed in gustatory receptor neurons, which encode seven transmembrane domains [77, 78]. GRs mainly respond to non-volatile compounds, including sugars, salts, gustatory pheromones, bitter compounds, and carbon dioxide [79–82]. The number of GRs was the highest in *P. micranthus* compared with the other three mirid bugs, and the number of GRs in phytophagous was more than carnivorous (Fig. 3b), consistent with the previous study [11]. However, the number of GRs identified differed due to different identification and screening methods. ORs were the first family of chemosensory receptors to be discovered in insect OSNs, and their function depends on the highly conserved odorant receptor co-receptor (Orco) gene. Orco can form Orco-ORx complexes with conventional olfactory receptors rather than odorant ligands to improve the efficiency of traditional olfactory receptor responses to odours [83, 84]. In this study, the *P. micranthus* genome contained 99 OR genes, including one Orco, which clustered in one branch with Orco of *A. lucorum* (Additional file 1: Fig. S8b). Moreover, like omnivorous *A. lucorum* and *H. halys*, non-omnivorous *P. micranthus* and *N. lugens* also had higher ORs numbers than other insects, indicating an apparent variance in ORs among different insects. IRs are a class of ionotropic glutamate receptors (iGluRs) and consist of two subfamilies: antennal IRs and divergent IRs [85, 86]. Unlike GRs and ORs, the primary receptor proteins for detecting odorants and tastants, IRs mainly detect chemo-, thermo-, and hygro-sensory stimuli [76, 82, 85]. There was little difference in the number of IRs among different species, which suggested that IRs are evolutionarily conserved (Fig. 3a). OBPs are a class of water-soluble proteins (approximately 150 amino acids) widely found in the olfactory mucosa of vertebrates and the sensilla fluid of insects [87, 88]. The first member of the OBP family was identified in the antennae of male *Antheraea polyphemus* (Lepidoptera: Saturniidae) [89]. OBPs are responsible for carrying odorant molecules to chemoreceptors located on sensory neurons, and OBPs may also be related to olfactory gene encoding and stimulus inactivation [88]. Based on their primary protein sequences and conserved cysteine number, OBPs have been classified into four subfamilies: classical OBPs, plus-C OBPs, minus-C OBPs, and atypical OBPs in Diptera or Lepidoptera [90, 91]. Only two subfamilies of classical and plus-C OBPs are present in Hemiptera; for example, 12 classical OBPs and two plus-C OBPs in *Adelphocoris lineolatus*, 20 classical OBPs and 12 plus-C OBPs in *L. lineolaris*, 24 classical OBPs and two plus-C OBPs in *Corythucha ciliata*, and 19 classical OBPs and three plus-C OBPs in *Yemma signatus* [92–95]. Our research identified 33 OBPs in the *P. micranthus* genome (Fig. 3a), including 17 classical OBPs and 16 plus-C OBPs (Additional file 1: Fig. S9, S10a). Moreover, phylogenetic analysis showed species-specific GR, OR, and OBP genes clustered in the same clades (Additional file 1: Fig. S8a, 8b, and 10a). The duplication of those chemosensory genes may be related to the ability of *P. micranthus* to recognize *M. micrantha* [96, 97] specifically.

In total, 34 lipases, 122 serine proteases (SPs), 20 polygalacturonases (PGs), 27 cysteine proteases (CPs), and five alpha-amylases were identified in the *P. micranthus* genome (Fig. 3b). Of these, SPs, PGs, and CPs were three significantly expanded gene families in *P. micranthus* (Fig. 2d). SP is widespread, including all kingdoms of cellular life and many viruses [96]. SPs are essential digestive enzymes in insects' physiological and pathological functions, such as fibrinolysis, development, fertilization, digestion, and immune defence [96, 97]. The main digestion-related functions of SPs are the breakdown of proteins into free amino acids and the degradation of plant toxins [22, 98]. Due to its diverse functionalities, the number of SPs in each species was significantly higher than that of other genes (Fig. 3b). PG is a vital cell hydrolysis enzyme. It causes visible plant injury, which catalyzes the hydrolysis of α-1,4-glycosidic linkages in polygalacturonic (pectic) acid in mirids, weevils, and a few other insect species [99–101]. PG has been widely described in fungi, bacteria, nematodes, and plants [102]. In insects, PGs have been reported in many orders with piercing-sucking and chewing mouthparts, beetles (Coleoptera, mainly of the Phytophaga clade) are included and notably common in mirid bugs (Hemiptera) [103–105]. Mirid PGs can cause much larger lesions than superficial mechanical damage or feeding by other sap-sucking insects [102]. Moreover, studies have found that microinjection of Lygus PG can cause cotton flower abortion [20]. On the contrary, many plants produce polygalacturonase-inhibiting proteins (PGIPs) to reduce insect PG activity [106]. Among 11 Hemiptera insects, PGs were identified only in four mirid bugs: *P. micranthus* (20), *A. lucorum* (52), *N. tenuis* (6), and *C. lividipennis* (1) (Fig. 3b). The food sources that insects can obtain determine the type and quantity of digestive enzymes [107]. The number of PGs in the *P. micranthus* genome was lower than that of the omnivorous plant bug *A. lucorum* and higher than that of the carnivorous *N. tenuis* and *C. lividipennis* (Fig. 3b). Furthermore, the phylogenetic analysis showed that the PGs of each mirid bug were primarily clustered in one species-specific clade, suggesting that PGs were evolutionarily conserved (Additional file 1: Fig. S10b). CP is an essential group of proteolytic enzymes in insects and has been reported in *Drosophila melanogaster*, *Tenebrio molitor*, *Tribolium castaneum*, and *Frankliniella occidentalis* [108–111]. Because CPs show better activity and stability at a slightly acidic pH (5–7), they are mainly found in the anterior midgut [112, 113]. CPs are associated with the hydrolysis of yolk proteins, protein turnover in lysosomes, and tissue decomposition [114]. There was a slight difference in the number of CPs between these species (Fig. 3b), it may be in part due to the function of CPs were diverse. However, the number of CPs in oligophagous species was generally lower than that of omnivorous species. Therefore, the low number of CPs in oligophagous species may be due to its narrow host range. Plants, like PGIPs, can synthesis cysteine peptidase inhibitors to inhibit CP activity [115, 116]. Based on this, *M. micrantha* can be controlled by RNA interference (RNAi) to inhibit the synthesis of PGIPs and cysteine peptidase inhibitors or to develop environment-friendly specific biological control agents.

**Fig. 4:**
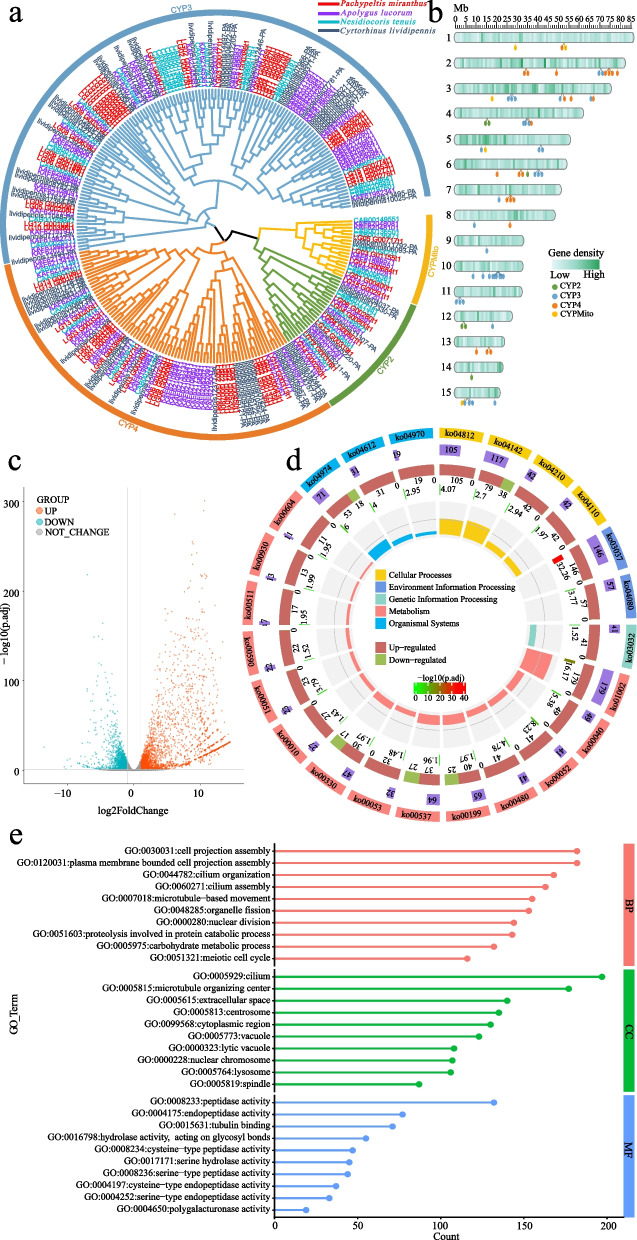
Cytochrome P450 genes in the *Pachypeltis micranthus* genome and the salivary gland transcriptome analysis of *P. micranthus*. **a** The maximum likelihood phylogenetic tree of P450 among four mirid bugs (*Pachypeltis micranthus*, *Apolygus lucorum*, *Nesidiocoris tenuis*, and *Cyrtorhinus lividipennis*). The outer circle indicates four main clades of P450. **b** Distribution of 69 *P. micranthus* P450 genes in the chromosome-level scaffolds. Gene density is shoed across the chromosome-level scaffolds by heat map. **c** Volcano plot shows the differentially expressed genes (DEGs) profiles among the salivary gland and the whole body of *P. micranthus*. **d** Diagram of KEGG pathway enrichment analysis (*p* < 0.05) of the significantly upregulated genes. From the outer to the inner circles. First circle: representation of the access number and classification of the KEGG Orthology (KO) group. Different colours represent different classifications. The second circle represents the total number of genes enriched in each KO group. The blocks of different lengths show the quantity information. Third circle: indication of the numbers of genes upregulated (red) and downregulated (olive green) in each KO group. The numbers below the colour block are upregulated and downregulated gene numbers. Forth circle represents the significance of enrichment analysis. The numbers next to the block and colour scale correspond to -log10(*p*-adjusted value, *p*.adj) of the significance of enrichment. Fifth circle: representation of the enrichment factor. The enrichment factor represents the ratio of the enrichment genes to background genes. **e** Gene Ontology (GO) enrichment analysis (*p* < 0.05) of the significantly upregulated genes. For easier visualization, only displayed the top ten GO terms for different aspects (BP: Biological process, CC: Cellular component, MF: Molecular function), respectively

Lastly, four important detoxification enzyme families were manually identified in the *P. micranthus* genome, including 31 GSTs, 69 P450s, 56 CCEs, and 44 ABCs (Fig. 3c). *P. micranthus* had the highest number of CCE genes and the second highest number of GST genes and P450 genes compared to the other four mirid bugs. The number of ABCs genes in *P. micranthus* is the second lowest and slightly higher than *N. tenuis* compared to the other hemipteran insects. These findings illustrated that *P. micranthus* might have a unique way of metabolizing toxic substances from food and the environment. Among these four detoxification gene families, as previously described, P450 was a significantly expanded gene family in *P. micranthus* that was annotated in the KEGG enrichment analysis (Fig. 2e). Cytochrome P450, or *CYP* genes, is one of the most prominent gene families, broadly distributed in nearly all living organisms [117, 118]. P450s are involved in the synthesis and metabolism of endogenous compounds and the metabolism of many exogenous compounds, such as a series of pesticides, hormones, steroids, fatty acids, and plant toxins [119, 120]. Therefore, P450s are crucial for insects to adapt successfully to their host plants [121]. According to the evolutionary relationships in the phylogenetic tree, insect P450s are divided into four clades: CYP2, CYP3, CYP4, and mitochondrial (Mito) [122, 123]. In total, 6 CYP2, 36 CYP3, 22 CYP4, and 5 Mito genes were identified in the *P. micranthus* genome, and the number of CYP3 genes was the largest (Fig. 4a). Phylogenetic analysis showed that many P450 genes were grouped in the CYP3 and CYP4 clades. Mapping of P450 genes to *P. micranthus* chromosome-level scaffolds showed that the CYP3 and CPY4 genes were mainly mapped to 2, 3, 6, and 10 chromosome-level scaffolds; in particular, chromosome-level scaffold 10 was only clustered many CYP3 genes (Fig. 4b). These results indicated that CYP3 genes experienced a relatively recent species-specific expansion in *P. micranthus*. CYP3 is pivotal in detoxifying plant secondary metabolites and pesticide resistance [124]. The expansion of CYP3 genes in *P. micranthus* might be associated with its specific detoxification of toxic substances of *M. micrantha* and evolutionary adaptation to *M. micrantha*.

### Salivary gland transcriptome analysis of *P. micranthus*

Following the assembly, 11,746 genes were generated using Hisat2 in the salivary gland transcriptome, consistent with the result in genome annotation (Table 2). Gene enrichment analysis showed that among the 11,746 genes, 7814 genes were associated with 7102 GO terms, and 6364 genes were associated with 437 KEGG Orthology (KO) terms.

To further understand the gene expression in the salivary gland, the expression of the genes in the salivary gland was then compared to the expression of the genes in the whole body. Genes with an absolute fold change equal to or greater than 2.0 and a *p*-adjusted value (*p*.adj) less than or equal to 0.05 were considered differentially expressed. Using these criteria, we obtained 1593 downregulated genes, 7015 not differentially expressed, and 2798 upregulated genes (Fig. 4c). The upregulated genes were more specific and highly expressed in the salivary gland than in the whole body. KEGG and GO enrichment analysis of the upregulated genes were further performed using clusterProfiler based on Evolutionary genealogy of genes: Non-supervised Orthologous Groups (EggNOG) v2.1.3 [125] annotations. KEGG enrichment analysis revealed that most of the upregulated genes were significantly (*p*.adj ≤ 0.05) involved in metabolism pathways, such as Peptidases and inhibitors (ko01002, contained179 genes), Protein digestion and absorption (ko04974, contained 53 genes), Galactose metabolism (ko00052, contained 41 genes), Glutathione metabolism (ko00480, contained 41 genes), Cytochrome P450 (ko00199, contained 40 genes) (Fig. 4d). Of interest, glutathione plays a vital role in plant disease resistance, cell proliferation, root development, salt tolerance, and cold injury protection [126]. The pathways of “Glutathione metabolism” and “Cytochrome P450” in insects were beneficial for inhibiting plant defence response and metabolizing and detoxifying xenobiotics from the plant [121]. In addition, 19 genes were significantly enriched in “Salivary secretion” (ko04970), and some enriched KEGG pathways contained downregulated genes (Fig. 4d). For GO enrichment analysis, for easier visualization, only displayed the top ten GO terms for different aspects (biological process, cellular component, and molecular function), respectively (Fig. 4e). Notably, nearly all Go terms were associated with peptidase activity in molecular function, especially among cysteine peptidase, serine peptidase, and polygalacturonase (Fig. 4e). The expanded gene families of *P. micranthus* also significantly enriched most of these GO terms (Fig. 2d). *Lygus linearis* salivary gland genes were also significantly enriched in those terms [18], which revealed a similar enrichment pattern of the two mirid bugs. However, apart from Miridae, even phytophagous Hemipteran belonging to the same family showed different gene enrichment patterns in salivary gland transcriptome, such as *Nephotettix cincticeps* (Cicadellidae) [127], *Nephotettix cincticeps* (Cicadellidae) [128], *Sogatella furcifera* (Delphacidae) [129], and *Bemisia tabaci* (Aleyrodidae) [130]. Therefore, these results may illuminate that phytophagous mirid bugs has a specific salivary enzyme system. Since the study of mirid bugs’ salivary glands was rare, this needs to be explored further. Additionally, the highly expressed genes in the *P. micranthus* salivary gland were significantly associated with metabolism pathway, peptidase activity, cysteine peptidase, serine peptidase, and polygalacturonase, which might also be a reason for precisely and highly efficiently feeding by *P. micranthus* on *M. micrantha*.

The salivary gland is a crucial organ in insects for secreting saliva [14], which contains a diverse array of effectors that actively suppress plant immune responses. Effectors such as C0002, Armet, Mp1, Mp2, Mp10, Me47, GroEL, migration inhibitory factor, and polygalacturonase (PG) have been identified in aphids, spider mites, and planthoppers [131, 132]. Among them, PG is also an important digestive enzyme [133], and its roles in the saliva of mirid bugs were reported forty years ago [134]. The PG in saliva can cause plant damage similar to that caused by mirid bugs, such as necrosis at the wound site, embryo abortion, and reduced plant growth [135, 136]. Injecting a solution containing purified PG extracted from Lygeus heads can cause damage to plants while injecting an equal volume of solution without PG does not produce any symptoms [20]. Therefore, PG is pivotal in the severe damage caused by mirid bugs to plants. Like other mirid bugs, *P. micranthus* feeding can trigger serve damage to *M. micrantha*, such as leaf discolouration, necrosis of the feeding site, organ abscission, flower bud abortion, and even the death of the entire plant [2, 23, 24]. This study found that PG was highly expressed in the salivary gland of *P. micranthus* and significantly enriched. Moreover, it was a significantly expanded gene family in the *P. micranthus* genome. Plants commonly rely on three signalling substances, salicylic acid (SA), jasmonic acid (JA), and ethylene (ET), to mediate plant immune responses [137]. The interactions of these signalling substances with other plant hormones, such as abscisic acid (ABA), gibberellins (GA), auxins (IAA), cytokinins (CK), and brassinosteroids (BR), are also essential in the plant’s response to external biotic stresses [138, 139]. The mouthparts of piercing-sucking insects cause relatively minor mechanical damage to plant tissues or cells, and the mode of damage is similar to that caused by pathogens. In response, plants primarily respond to such damage through the SA signalling transduction pathway [140]. Insect feeding can also suppress plant growth-related counterparts [141]. Consequently, the PG of *P. micranthus* may induce alterations in plant growth and development-related hormones or substances, leading to the inhibition of growth and development and flower sterility in *M. micrantha*. Furthermore, PGs were relatively conserved across species (Additional file 1: Fig. S10b). Hence, it is possible to further screen *P. micranthus* PG with specific control capabilities against *M. micrantha* and develop environmentally friendly, safe, and specific RNA interference (RNAi) herbicides based on shRNA [142] for the targeted control of *M. micrantha*.

## Conclusions

This work has combined the MGISEQ-2000–, Nanopore–, and Hi-C technologies to generate high-quality chromosome-level scaffolds of *P. micranthus*, a vital biocontrol agent for *M. micrantha* (a malignant invasive weed widely distributed worldwide). Genomic structure and functional annotation analyses showed high levels of completeness and continuity in the assembled genome. The *P. micranthus* genome size was 712.72 Mb with a contig N50 of 16.84 Mb, which included 15 chromosome-level scaffolds (707.51 Mb, 99.27% of the genome). The phylogenetic analysis indicated that *P. micranthus* and two other mirid bugs (*A. lucorum* and *N. tenuis*) diverged from the common ancestor approximately 200 million years ago. In the *P. micranthus* genome, several gene families potentially associated with host adaptation had also been identified, including chemosensory, digestive, and detoxification gene families. In addition, when compared with the whole body, the salivary gland had a lot of upregulated genes associated with highly efficient feeding, which were more specific and highly expressed in the salivary gland. These genes would be helpful in the search for novel strategies to control *M. micrantha,* such as RNAi and gene editing. As an efficient biological agent to control *M. micrantha*, the high-quality genome provides significant resources for examining the evolutionary adaptation between mirid bugs and their hosts. It also contributes to improving the practical application and achieving large-scale artificial breeding of this mirid bug.

## Methods

### Sample collection and sequencing

*P. micranthus* final instar nymphs were collected in October 2020 on the leaves of *Mikania micrantha* at the edge of the reservoir near Ruili city in Yunnan Province, China. The insects were reared on *M. micrantha* leaves under a temperature of 24 ± 1℃, relative humidity of 70 ± 5%, and photoperiod of 14:10-h light: dark. Male adults (Fig. 1a_IV) were immediately frozen in liquid nitrogen to avoid interference from feeding and upon emergence.

The DNA samples used for different sequencing platforms were extracted separately following the manufacturer's protocol. High-quality genomic DNA was extracted from male adult bugs using the QIAGEN Genomic Kit (QIAGEN, MA, USA). DNA quality and concentrations were checked using agarose gel electrophoresis and Nanodrop. After purifying, sequencings were performed using Illumina, PacBio, and Nanopore sequencing platforms. The obtained reads sequenced from Illumina and PacBio were used for genome assembly correcting.

The library was prepared using the Ligation Sequencing Kit (Oxford Nanopore Technologies, MA, USA) according to the manufacturer’s instructions for Nanopore sequencing. Library quantification was done using Qubit 3.0 Fluorometer (Invitrogen, CA, USA). The sequencing was performed on the Nanopore GridION X5 sequencer. Nanopore reads were base-called using Guppy v3.2.2 [143] with quality filtering ( mean_qscore_template ≥ 7) to generate pass reads. The pass reads were used for subsequent genome assembly. For PacBio sequencing, library preparation was done using Sequel Sequencing Kit 2.0 (Pacific Biosciences, CA, USA). The library was sequenced on a PacBio Sequel II instrument to generate Circular Consensus Sequencing (CCS) reads. All procedures were carried out according to the manufacturer’s recommendations. The library preparation was done using a TruSeqDNA PCR-Free kit for Illumina sequencing according to the manufacturer’s protocols. The sequencing runs were performed using a BGI MGISEQ-2000 sequencing platform (BGI, Shenzhen, China). Raw reads from PacBio and Illumina were filtered to ensure reliability and accuracy using fastp v0.20.0 [144].

Hi-C library was constructed following a standard procedure [145]. Ten male adult insects were cut into pieces and then vacuum infiltrated in nuclei isolation buffer supplemented with 2% formaldehyde. Cross-linked DNA was digested with DpnII. Chromatin ends were marked with biotin-14-dCTP and ligated by T4 DNA polymerase. DNA was sheared into 300–600 bp fragments, and the fragments were blunt-end repaired and A-tailed, followed by purification through biotin-streptavidin-mediated pull-down. The adapters were ligated to the Hi-C products, and then the resulting Hi-C library was amplified using polymerase chain reaction (PCR). Finally, the Hi-C library was quantified and sequenced using an Illumina Novaseq platform (Illumina, CA, USA).

For transcriptome sequencing, three male adults were washed with 95% ethanol three times to avoid surface microbial contamination from the body surface. In the previous study [22], salivary glands were dissected from the head-prothorax. A total of 50 salivary glands were collected. Three biological replications were set for the sample. RNA sequencing libraries were prepared using the Illumina TruSeq stranded RNA Library Prep Kit (Illumina) following the manufacture’s protocols. The resulting libraries were performed on an Illumina HiSeq sequencer (Illumina, CA, USA).

### Transcriptome assembly and analysis

RNA sequencing raw data were filtered by removing adapter sequences and low-quality reads using fastp v0.20.0 [144]. The clean reads were assembled to the *P. micranthus* genome using Hisat2 v2.1.0 [146]. Gene expression levels were quantified as fragments per kilobase of transcript per million mapped reads (FPKM) using StringTie [147]. The expression count of each gene from the assembled genome was generated using the HTSeq package v2.0.2 [148]. DESeq2 v1.38.3 [149] was used to analyze the differential expression, and the analysis was performed in R v4.2.2 (https://www.r-project.org/). Differentially expressed genes screening conditions were set to false discovery rate (FDR)-adjusted *p*-value < 0.05 and fold change ≥ 2. EggNOG annotations were obtained using eggNOG-Mapper v2.1.3 with HMM search mode [125]. Then Kyoto Encyclopedia of Genes and Genomes (KEGG; www.genome.jp/kegg/) [70] and Gene Ontology (GO; Gene Ontology Consortium, geneontology.org) [61] enrichments were analyzed using the R package clusterProfiler v4.6.2 based on the eggNOG annotations.

### Genome size and heterozygosity

Before assembly of the *P. micranthus* genome, genome size and heterozygosity were evaluated using a k-mer analysis. Illumina short reads were filtered and used to estimate the distribution of 17-mer frequency with jellyfish program v2.3.0 [150]. Subsequently, the k-mer results were fitted and analyzed using the skew-normal distribution model and the negative binomial model. The genome size and heterozygosity were estimated by the corresponding software FindGSE v1.94 [119] and GenomeScope v1.1.1 [120], respectively, based on the two algorithms. Genome size was evaluated using the following equation: genome size = k-mer number/peak depth. To obtain a more accurate estimate of the heterozygosity rate, the genome of *Arabidopsis thaliana* was employed to simulate the expected depth of short-read data, followed by fitting the k-mer curve under various gradient heterozygosity rates. The heterozygosity rate was subsequently determined based on the fitting of the k-mer curve. Then, the heterozygosity and repeat content of *P. micranthus* were assessed by combining the different simulation data of *Arabidopsis* heterozygosity and distribution of 17-mer frequency.

### Genome assembly and assessment of assembly quality

De novo genome assembly of Nanopore long reads was done using NextDenovo package v2.3.1 (read_cutoff = 1 k, seed_cutoff = 29 k). Firstly, the primary correction was performed using the NextCorrect module to obtain consistent sequences (CNS reads). The CNS reads were then used for preliminary assembly with the NextGraph module. Finally, to improve the mean accuracy of bases, the initially assembled contigs were polished with several rounds of correction [151] [PacBio long reads (three rounds) and Illumina short reads (four rounds)] using NextPolish v1.3.0 [51] to obtain the polish genome.

We used Benchmarking Universal Single-Copy Orthologs (BUSCO) v4.0.5 [52] and CEGMA v2 [53] to assess completeness to evaluate the genome assembly quality. Illumina short reads were mapped onto the genome using BWA (Burrows-Wheeler Aligner) v0.7.12 [54] and mem algorithm with defaults parameters, and the mapping rate and genome coverage of sequencing reads were calculated using samtools v1.4 [152] to assess the accuracy and consensus of the assembled genome. Furthermore, the assembled genome base accuracy was calculated using BCFtools v1.8.0 [153]. For the GC-depth analysis, Nanopore long reads were mapped onto the genome assembly using Minimap2 v2.24 [154], and the GC content and the reads coverage were calculated for each sliding window (size of 10 kb). All the RNA-seq reads were aligned against the assembly using Hisat2 v2.1.0 [146] to evaluate the coverage of expressed genes of the assembly. Finally, to examine the interspecies contamination, the assembled genome was divided into 1 Mb bins and aligned with the sequences from the nucleotide sequence database (NT, ftp.ncbi.nih.gov/blast/db, downloaded March 1st 2021) using BLASTN [155].

### Scaffolding with Hi-C

To generate the chromosome-level scaffolds of *P. micranthus*, the Hi-C reads were used to detect the scaffold contact information for assisting genome assembly. Briefly, raw Hi-C paired-end reads were filtered out by removing adapter sequences, and low-quality reads using fastp v0.20.0 [144]. The cleaned reads were aligned to the draft genome sequence using Bowtie2 v2.3.2 with strict paraments (-L 30) [156] to obtain unique mapped paired-end reads. According to the Hi-C protocol, Hi-C-Pro v2.8.1 [157] was used to further duplication remove, sort, and quality assessment to obtain preliminary chromosome-level scaffolds contact maps. The assembly package, LACHESIS (https://github.com/shendurelab/LACHESIS), was used to cluster, order, and orient scaffolds onto chromosome-level scaffolds [158]. Finally, the predicted chromosome-level scaffolds were cut into 100 kb bins and built heatmap according to the interaction signals revealed by mapped Hi-C read pairs between bins.

Genomic collinearity blocks for intra-species of *P. micranthus* were identified using MCScanX [159] software with default parameters. The intra-species collinearity analysis and genome annotation results were visualized using Circos v0.69–8 [160]. Synteny of the *P. micranthus* genome with the *A. lucorum* and *C. lividipennis* genomes were analyzed and visualized using JCVI [57] (the Python version of MCScanX) to identify chromosome-level scaffolds structural changes among the three mirid bugs.

### Genome annotation

Homology-based and de novo methods were used to annotate transposable elements (TEs) in the *P. micranthus* genome. For the de novo method, the repeat library of *P. micranthus* was identified and constructed using RepeatModeler v1.0.11 with default parameters [161]. And then, long terminal repeats and miniature inverted-repeat transposable elements (MITEs) identification were performed using LTR_FINDER and MITE-Hunter [162, 163]. RepeatMasker v1.331 [130] was used for the homology-based method to predict repeat sequences in the *P. micranthus* genome by searching against the Repbase [164] and de novo repeat libraries.

The protein-coding genes of *P. micranthus* were annotated using homolog searching, transcriptome sequencing, and de novo prediction. For homolog searching, the protein sequences from six Hemiptera insects (*A. lucorum*, *N. tenuis*, *A. gossypii*, *N. lugens*, *D.*, and *H. halys*) were downloaded from NCBI to align to the *P. micranthus* genome sequence using BLAST v2.7.1 [155]. Then, the high-similarity sequences were filtered using GeMoMa v1.6.1 [165] to obtain the gene structure. In transcriptome-based analysis, the RNA sequencing data, as described above, was aligned to the *P. micranthus* genome using STAR v2.7.3 [166]. Augustus v3.3.1[167] with default parameters was used to perform de novo predictions with the training set. Finally, all predicted genes from the above three methods were integrated using EVidenceModeller v1.1.1 [168] to generate a final nonredundant gene set in which genes with TEs were removed using the TransposonPSI package (http://transposonpsi.sourceforge.net/), and the miscoded genes were further filtered. Using PASA, the RNA sequencing assemblies were employed to determine untranslated regions (UTRs) and alternative splicing regions. The longest transcripts for each locus remained, and the regions outside the open reading frames (ORFs) were designated UTRs.

To annotate the gene functions in the *P. micranthus* genome, the official gene set was aligned to five databases using BLASTP v2.7.1 with an e-value of 1e-5. The five databases were: NCBI non-redundant amino acid sequences (NR), KEGG, Cluster of Orthologous Groups for eukaryotic complete genomes (KOG), GO, and Swissprot database.

Five types of ncRNAs, rRNA, snRNA, miRNA, regulatory RNAs, and tRNA, were annotated. The tRNAs were identified using tRNAscan-SE v2.0 [169] with eukaryote parameters. The rRNAs and their subunits were predicted using RNAmmer v1.2 [170]. The snRNAs and miRNAs fragments in *P. micranthus* were detected by aligning against the Rfam database (release 14.0) [171] using Infernal v1.1.2 [172].

All software, versions, and parameters used for genome assembly and annotation were provided in Additional file 2 (Table S24).

### Phylogenetic analysis

Protein sequences of nine published whole-genome species (*N. tenuis*, *A. lucorum*, *A. gossypii*, *N. lugens*, *D. citri*, *H. halys*, *A. glycines*, *R. maidis,* and *C. lectularius*) were downloaded from NCBI. TBtools v1.045 [173] was used to extract the longest transcript of each gene based on the total length of coding sequences (CDS). Additionally, genes with erroneous coding and those exhibiting premature termination were discarded. The extracted protein sequences were aligned pair-wise to search conserved orthologs using BLASTP v2.7.1 with an e-value of 1e-5. OrthoMCL v2.0.9 [60] with default parameters was used to cluster gene families. And then, the single-copy genes were multiple-aligned using MAFFT v7.313 [174]. The poorly aligned sequences and ambiguous regions were removed using Gblocks v0.91b [175], and a phylogenetic tree was constructed using RAxML v8.2.10 under a GTRGAMMA substitution model with 1000 bootstrap iterations. Furthermore, divergence time was estimated using the MCMCTree program in PAML v4.8 [176] based on the constructed polygenetic tree. Calibration time was obtained from articles and TimeTree (http://www.timetree.org/) database (Additional file 2: Table S25). Expansion and contraction of orthologous genes were analyzed using CAFE v4.2.1 [63], which uses a birth–death process to model gene gain and loss over a phylogeny.

Positive selection can be inferred from a higher ratio of nonsynonymous substitution (d_N_) over the synonymous substitution (d_S_) per site (d_N_/d_S_ > 1) [177]. In this analysis, the single-copy genes of *P. micranthus* were used to calculate average d_N_/d_S_ values and conducted the branch-site likelihood ratio test using the CodeML program in PAML package v4.8 [176]. Genes were considered positively selected genes if *p*-value < 0.05 under the branch-site model.

### Manual annotation of candidate gene family

Manual identification was performed using BLASTP and hmm software for candidate gene family annotation. First, the set of reference protein sequences of each gene family was downloaded from NCBI and aligned against the *P. micranthus* protein set using BLASTP v2.2.31 with an e-value of 1e-5 to search significant hists. Further, Hmmsearch [178] was used to predict gene families in conjunction with the hmm model from the Pfam database [179]. For the gene family without hmm model in the Pfam database, the protein sequences downloaded from NCBI of each gene family were aligned using MUSCLE v3.8.1551 [180], and hmm models were built using Hmmbuild. The significant hits (e-value of 1e-5) from BLASTP and Hmmsearch were merged, de-replicated, and filtered to generate final hits. The resulting hits were further verified and filtered using Conserved Domain Database (CDD) (http://www.ncbi.nlm.nih.gov/Structure/cdd/wrpsb.cgi) and Simple Modular Architecture Research Tool (SMART) (http://smart.embl-heidelberg.de/) databases by removing the sequences without domain. In addition, the sequences coding for fewer than 80 amino acids were discarded, and only the longest transcript was kept when multiple transcripts were identified to the gene.

For the gene family polygenetic analysis, protein sequences of each gene family were aligned using MAFFT v7.310 [181] and filtered using trimAl v1.4 [182] to obtain conserved blocks. Polygenetic tree inference was performed using Fasttree v2.1.11, and the resulting phylogenetic trees were visualized using Evolview (https://www.evolgenius.info/evolview/). The distribution of genes in chromosome-level scaffolds was mapped and visualized using the R package RIdeogram [183].

## Supplementary Information

**Additional file 1:** **Fig. S1.** Estimate of the Pachypeltis micranthus genome size with 17-mer. **Fig. S2.** The distribution of accumulated contigs length of Pachypeltis micranthus. **Fig. S3.** The GC-depth distribution of Nanopore data. **Fig. S4.** Chromosome-level scaffolds synteny between Apolygus lucorum and Cyrtorhinus lividipennis. **Fig. S5.** Characteristics of the annotated protein-coding genes in the Pachypeltis micranthus genome. **Fig. S6.** Timing of inferred divergence of 10 Hemiptera species. **Fig. S7.** Gene ontology (GO) enrichment analysis of species-specific genes of Pachypeltis micranthus. **Fig. S8.** Phylogenetic analysis of three chemoreceptor genes among Pachypeltis micranthus, Apolygus lucorum, Cyrtorhinus lividipennis, and Halyomorpha halys. **Fig. S9.** Sequence alignment of amino acids of Pachypeltis micranthus odorant-binding proteins (OBPs). **Fig. S10.** Phylogenetic analysis of odorant-binding proteins (OBPs) and polygalacturonases (PGs) among Pachypeltis micranthus, Apolygus lucorum, Cyrtorhinus lividipennis, and Halyomorpha halys.

**Additional file 2:** **Table S1.** Statistical information of 17-kmer analysis for the Pachypeltis micranthus genome. **Table S2.** Statistics of the genome assembly of Pachypeltis micranthus. **Table S3.** BUSCO scores of the assembled Pachypeltis micranthus genome. **Table S4.** CEGMA assessment results. **Table S5.** Statistics of the mapping rates on the genome assembly for Illumina reads. **Table S6.** Statistic of the accuracy of single-base in the assembled genome. **Table S7.** Statistics of the mapping rates on the genome assembly for Hi-C sequencing data. **Table S8.** Statistics of the valid paired-end reads of unique mapped paired-end reads. **Table S9.** Statistics of chromosome-level scaffolds in Pachypeltis micranthus genome assembly. **Table S10.** Statistics of repeat sequences identified in Pachypeltis micranthus genome. **Table S11.** Summary of protein-coding genes annotation of the genome assembly. **Table S12.** Comparative statistics of protein-coding genes between Pachypeltis micranthus and other Hemiptera species. **Table S13.** Statistics for functionally annotated protein-coding genes. **Table S14.** BUSCO assessment of the protein-coding genes. **Table S15.** Summary statistics for non-coding RNAs. **Table S16.** Conserved orthologs of ten species. **Table S17.** Statistics of species-specific genes in Pachypeltis micranthus genome. **Table S18.** KEGG enrichment analyses of the expanded gene families in the Pachypeltis micranthus genome. **Table S19.** GO enrichment analyses of the expanded gene families in the Pachypeltis micranthus genome. **Table S20.** GO enrichment analyses of the significantly contracted gene families in the Pachypeltis micranthus genome. **Table S21.** KEGG enrichment analyses of the significantly contracted gene families in Pachypeltis micranthus genome. **Table S22**. KEGG enrichment analysis of positively selected genes in Pachypeltis micranthus. **Table S23.** GO enrichment analysis of positively selected genes in Pachypeltis micranthus. **Table S24.** The software, versions, and parameters used for genome assembly and annotation. **Table S25.** Calibrating time for estimating divergence times (Million years ago, Mya).

## Data Availability

The genome data have been submitted to NCBI Sequence Read Archive (SRA) database under BioProject PRJNA755865. The Whole Genome Shotgun project was deposited at DDBJ/ENA/GenBank under the accession JAINFA010000000. The version described in this paper is the first version, JAINFA010000000. In addition, the genome annotation information is available on the DRYAD database https://datadryad.org/stash/share/7t-r6CBvOv5C8vuLqquY-FazPfGOPGkMyk7f_qqCoVk.

## Abbreviations

ABC: ATP-binding cassette transporter
BGI: Beijing Genomics institution
BUSCO: Benchmarking universal single-copy orthologs
BWA: Burrows-Wheeler Aligner
CCE: Carboxylesterase
CDD: Conserved Domain Database
CDS: Coding sequence
CEGMA: Core Eukaryotic Genes Mapping Approach
CNS: Consistent sequences
CP: Cysteine protease
CSP: Chemosensory protein
CYP: Cytochrome P450
EggNOG: Evolutionary genealogy of genes Non-supervised Orthologous Groups
FPKM: Fragments per kilobase of transcript per million mapped reads
GO: Gene Ontology
GR: Gustatory receptor
GST: Glutathione S-transferase
Gb: Giga bp
Hi-C: Chromosome Conformation Capture
IR: Ionotropic receptor
KEGG: Kyoto Encyclopedia of Genes and Genomes
KO: KEGG Orthology
KOG: Cluster of Orthologous Groups for eukaryotic complete genomes
Kb: Kilo bp
LINE: Long interspersed nuclear element
MITE: Miniature inverted-repeat transposable element
Mb: Million bp
Mya: Million years ago
NCBI: National Center for Biotechnology Information
NR: NCBI non-redundant amino acid sequences
NT: Nucleotide sequence database
OBP: Odorant-binding protein
OR: Odorant receptor
ORF: Open reading frame
OSN: Olfactory sensory neurons
Orco: Odorant receptor co-receptor
PCR: Polymerase chain reaction
PG: Polygalacturonase
PGIP: Polygalacturonase-inhibiting protein
PacBio: Pacific Biosciences
RNAi: RNA interference
SMART: Simple Modular Architecture Research Tool
SP: Serine protease
TE: Transposable element
TR: Tandem repeat
UTR: Untranslated region
d_N_/d_S_: Ratio of nonsynonymous substitution (d_N_) over the synonymous substitution (d_S_) per site
iGluRs: Ionotropic glutamate receptors
miRNAs: MicroRNAs
ncRNAs: Non-coding RNAs
*p*.adj: *P*-adjusted
rRNAs: Ribosomal RNAs
snRNAs: Small nuclear RNAs
tRNAs: Transfer RNAs
