# Supplementary material for: High-quality chromosome-level scaffolds of the plant bug Pachypeltis micranthus provide insights into the availability of Mikania micrantha control
Source: BMC Genomics. 2023 Jun 20;24:339. doi: 10.1186/s12864-023-09445-8 (PMC10280852; doi:10.1186/s12864-023-09445-8)
Supplement: Supplementary file 1 — Additional file 1: Fig. S1. Estimate of the Pachypeltis micranthus genome size with 17-mer. Fig. S2. The distribution of accumulated contigs length of Pachypeltis micranthus. Fig. S3. The GC-depth distribution of Nanopore data. Fig. S4. Chromosome-level scaffolds synteny between Apolygus lucorum and Cyrtorhinus lividipennis. Fig. S5. Characteristics of the annotated protein-coding genes in the Pachypeltis micranthus genome. Fig. S6. Timing of inferred divergence of 10 Hemiptera species. Fig. S7. Gene ontology (GO) enrichment analysis of species-specific genes of Pachypeltis micranthus. Fig. S8. Phylogenetic analysis of three chemoreceptor genes among Pachypeltis micranthus, Apolygus lucorum, Cyrtorhinus lividipennis, and Halyomorpha halys. Fig. S9. Sequence alignment of amino acids of Pachypeltis micranthus odorant-binding proteins (OBPs). Fig. S10. Phylogenetic analysis of odorant-binding proteins (OBPs) and polygalacturonases (PGs) among Pachypeltis micranthus, Apolygus lucorum, Cyrtorhinus lividipennis, and Halyomorpha halys. [file 12864_2023_9445_MOESM1_ESM.docx]

**Supplementary Materials**

**High-quality chromosome-level scaffolds of the plant bug *Pachypeltis micranthus* provide insights into the availability of *Mikania micrantha* control**

Xiafei Wang^1^, Ning Zhao^1^, Liqiong Cai^2^, Naiyong Liu^1^, Jiaying Zhu^1^, Bin Yang^1*^

^1^Key Laboratory of Forest Disaster Warning and Control of Yunnan Province, Southwest Forestry University, Kunming, China

^2^Key Laboratory for Forest Resources Conservation and Utilization in the Southwest Mountains of China, Ministry of Education, Southwest Forestry University, Kunming, China

*** Correspondence:** [yangbin48053@163.com](mailto:yangbin48053@163.com)

**Supplemental Figures**

**

**

**Fig. S1** Estimate of the *Pachypeltis micranthus* genome size with 17-mer. The clean data of short reads were used for the k-mer frequency calculation. The peak depth of this curve was 31. The estimated *P. micranthus* genome size was 708.32 Mb.


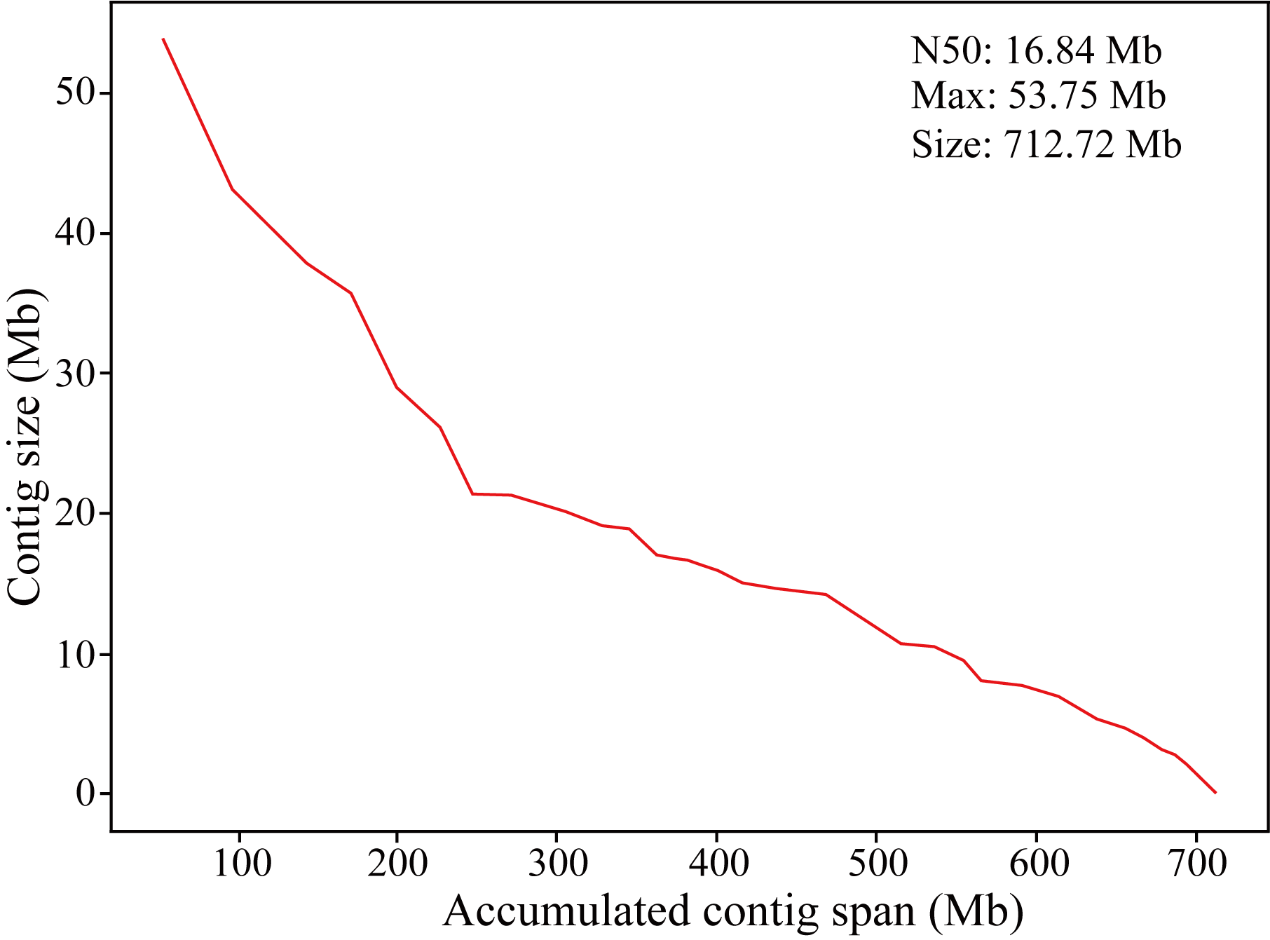


**Fig. S2** The distribution of accumulated contigs length of *Pachypeltis micranthus*.

**
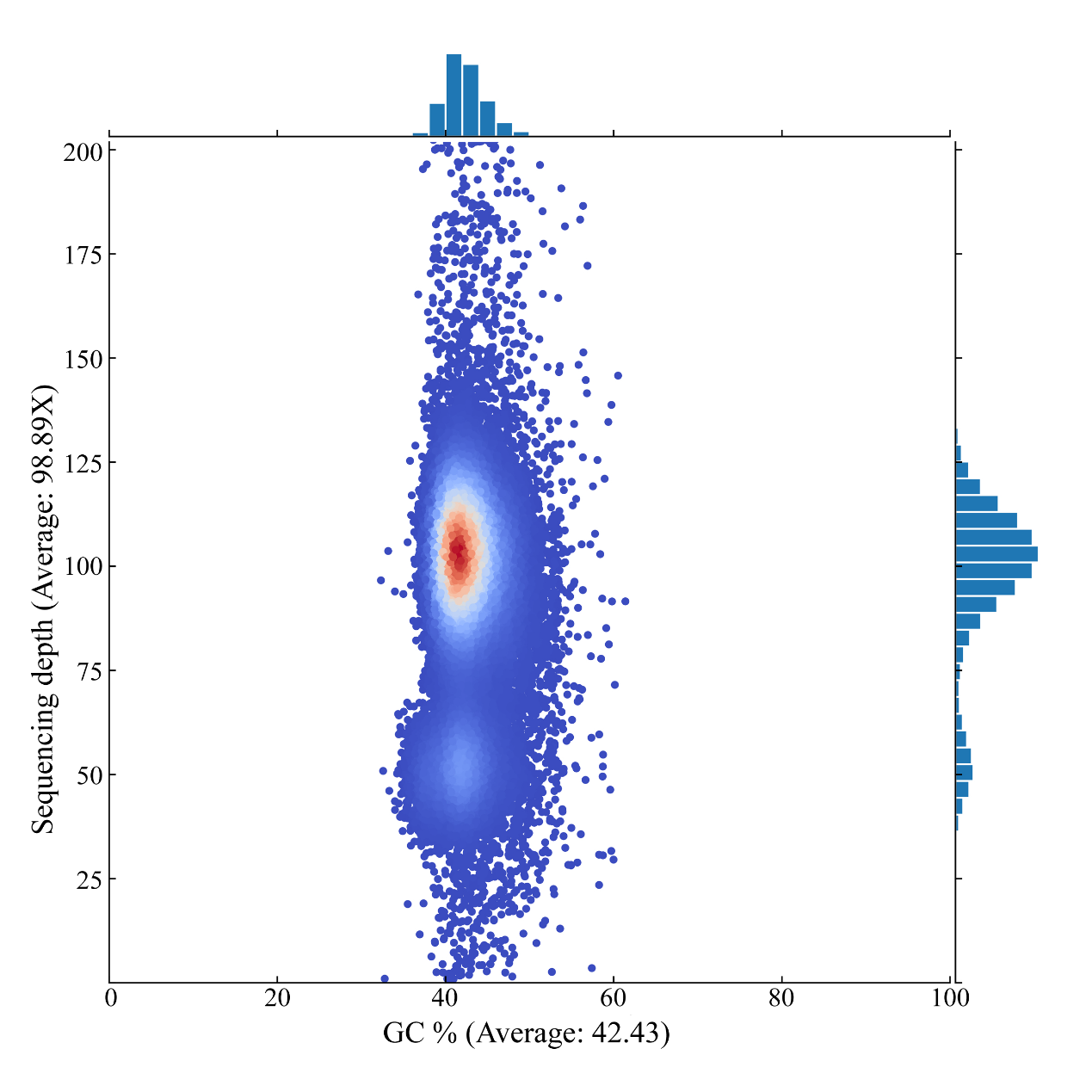
**

**Fig. S3** The GC-depth distribution of Nanopore data. Each contig's average GC content and depth in Nanopore data were counted using a 10 Kb sliding window size.

**
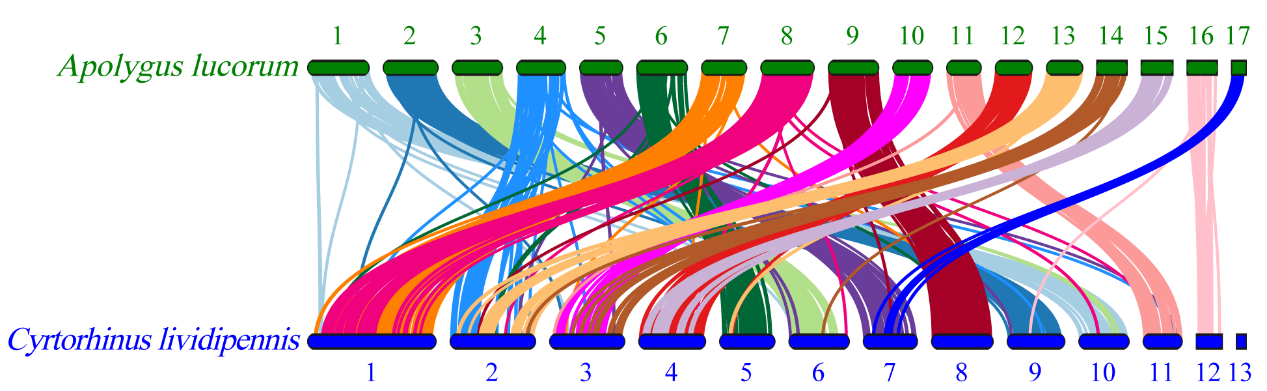
**

**Fig. S4** Chromosome-level scaffolds synteny between *Apolygus lucorum* and *Cyrtorhinus lividipennis*.

**
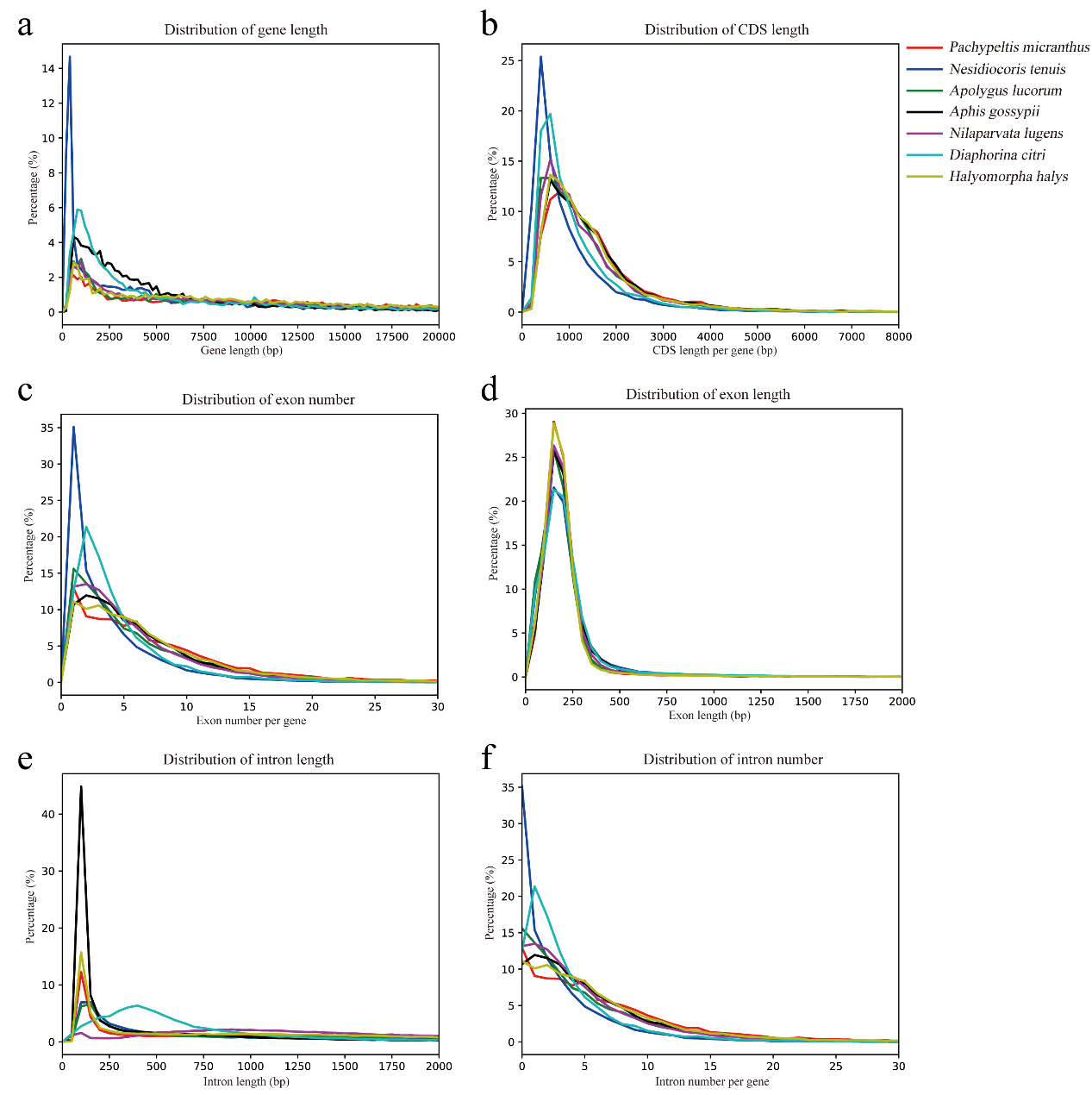
**

**Fig. S5** Characteristics of the annotated protein-coding genes in the *Pachypeltis micranthus* genome. (a) Distribution of the lengths of the protein-coding sequences. (b) Distribution of the lengths of CDSs. (c) Distribution of exon number. (d) Distribution of exon lengths. (e) Distribution of intron lengths. (f) Distribution of intron numbers.

**
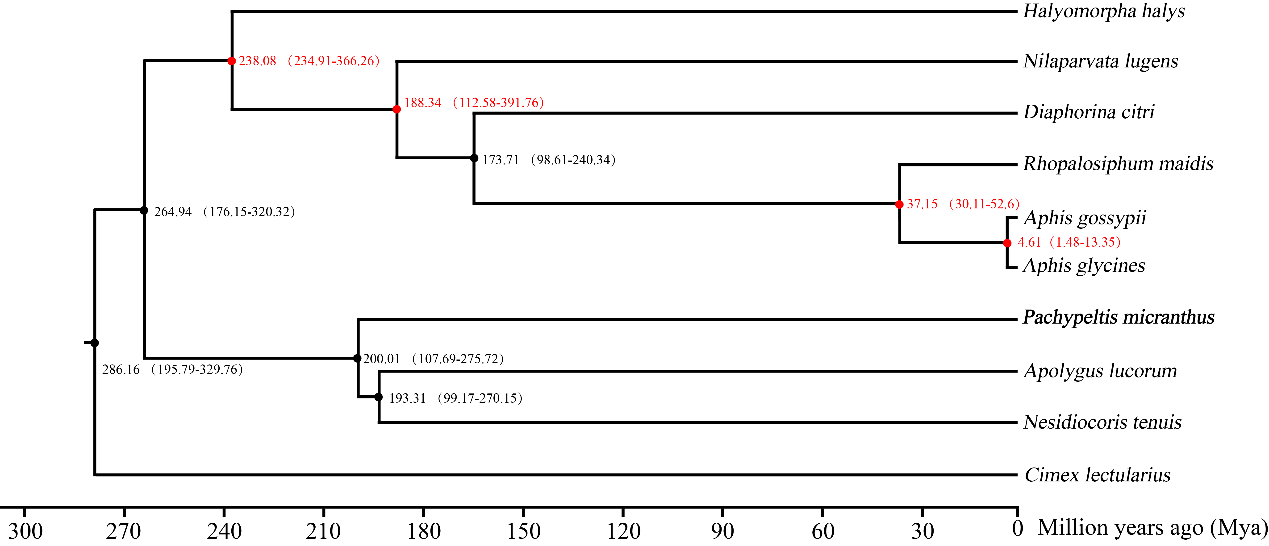
**

**Fig. S6** Timing of inferred divergence of 10 Hemiptera species. Numbers on the nodes indicate divergence time. The time calibration is illuminated as red dots in the tree.

**
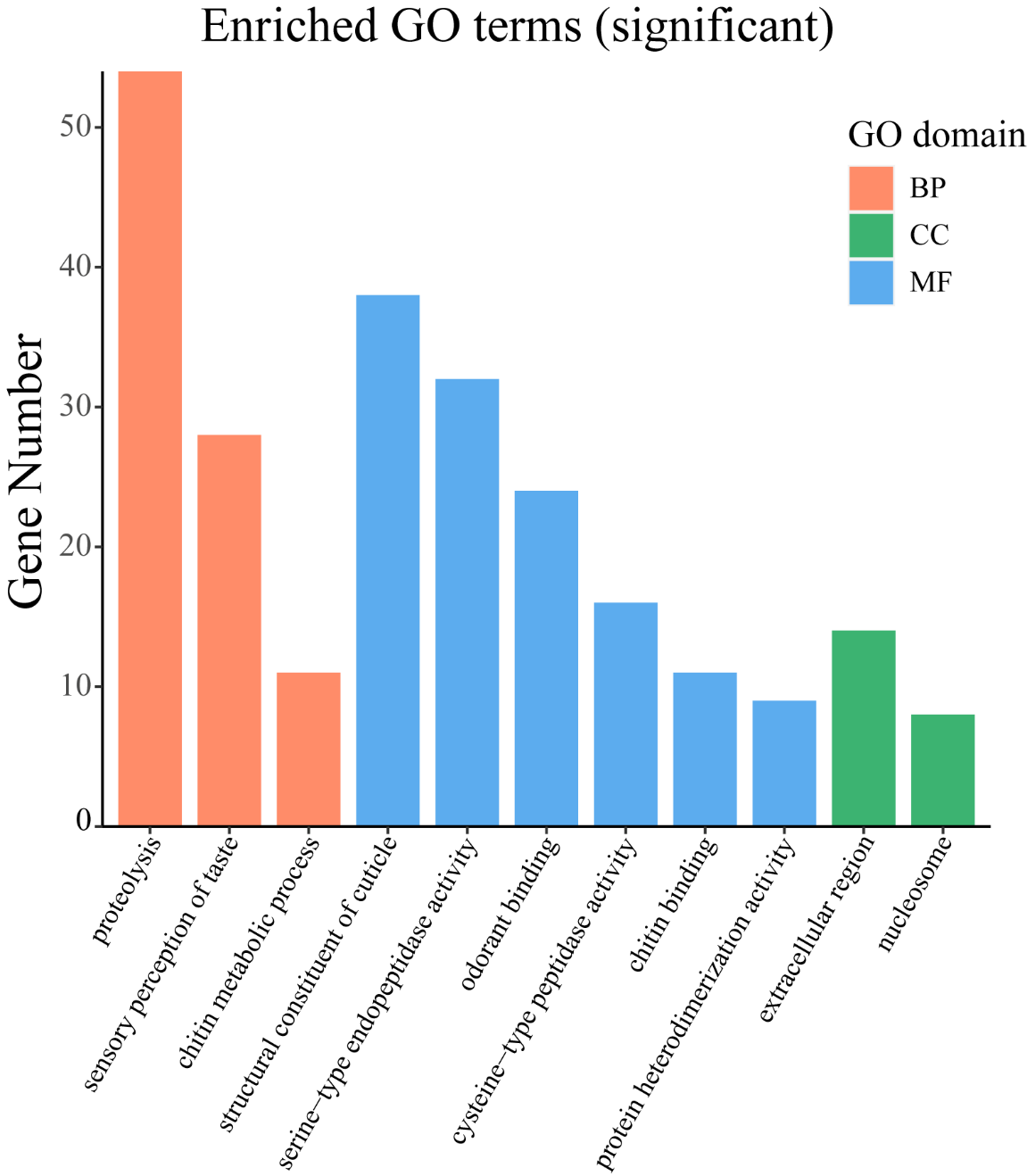
**

**Fig. S7** Gene ontology (GO) enrichment analysis of species-specific genes of *Pachypeltis micranthus*. The top 11 most significant GO categories were shown (*p* < 0.05)—BP, biological process; CC, cellular component; MF, molecular function.

**
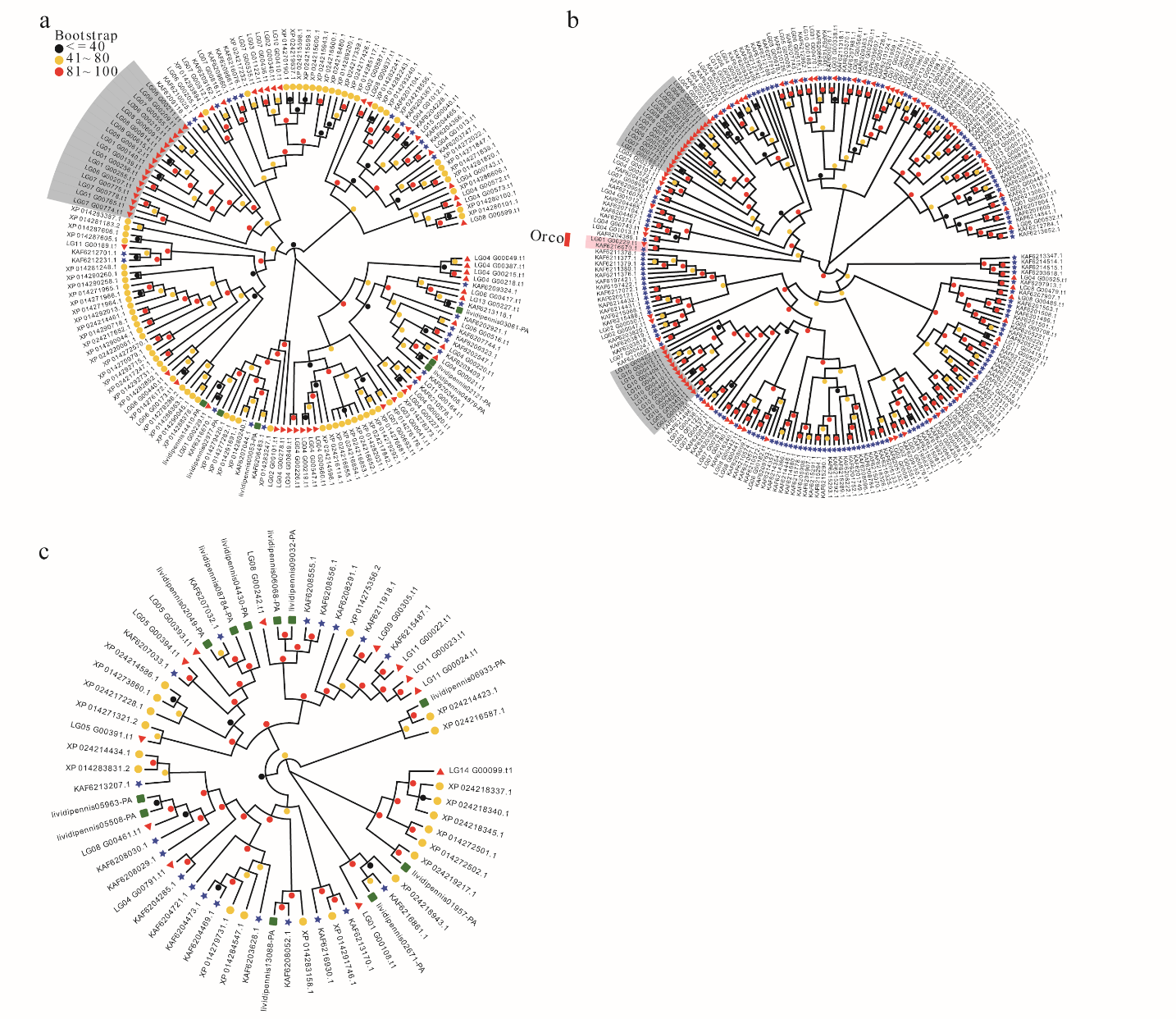
**

**Fig. S8** Phylogenetic analysis of three chemoreceptor genes among *Pachypeltis micranthus*, *Apolygus lucorum*, *Cyrtorhinus lividipennis*, and *Halyomorpha halys*. (a) gustatory receptor (GR); (b) odorant receptor (OR), odorant receptor co-receptor (Orco) genes are marked with pink shading; and (c) ionotropic receptor (IR). Species-specific expanded clades in *P. micranthus* are marked with grey shading.

**
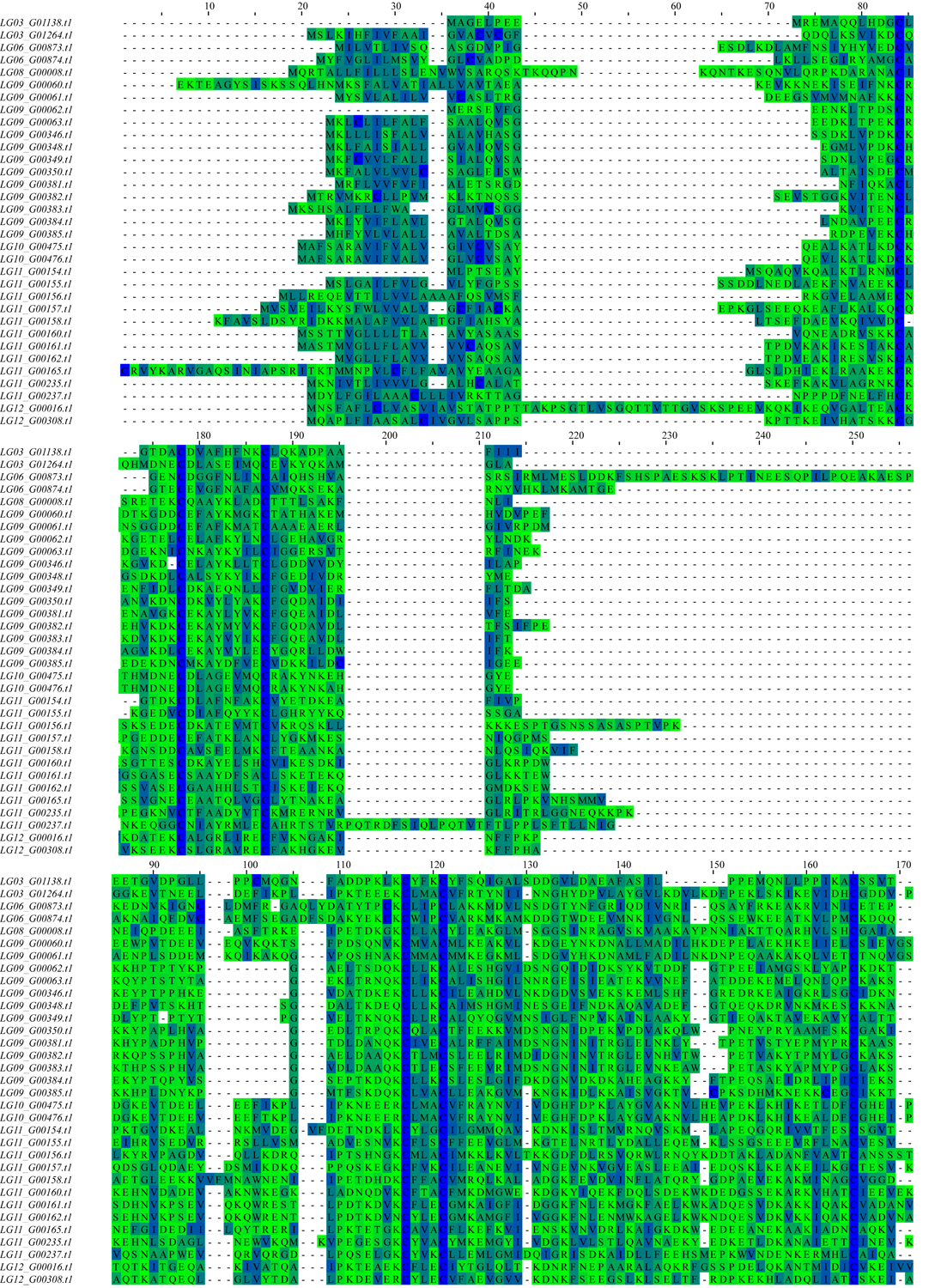
**

**Fig. S9** Sequence alignment of amino acids of *Pachypeltis micranthus* odorant-binding proteins (OBPs). Conserved cysteines are boxed in dark blue.

**
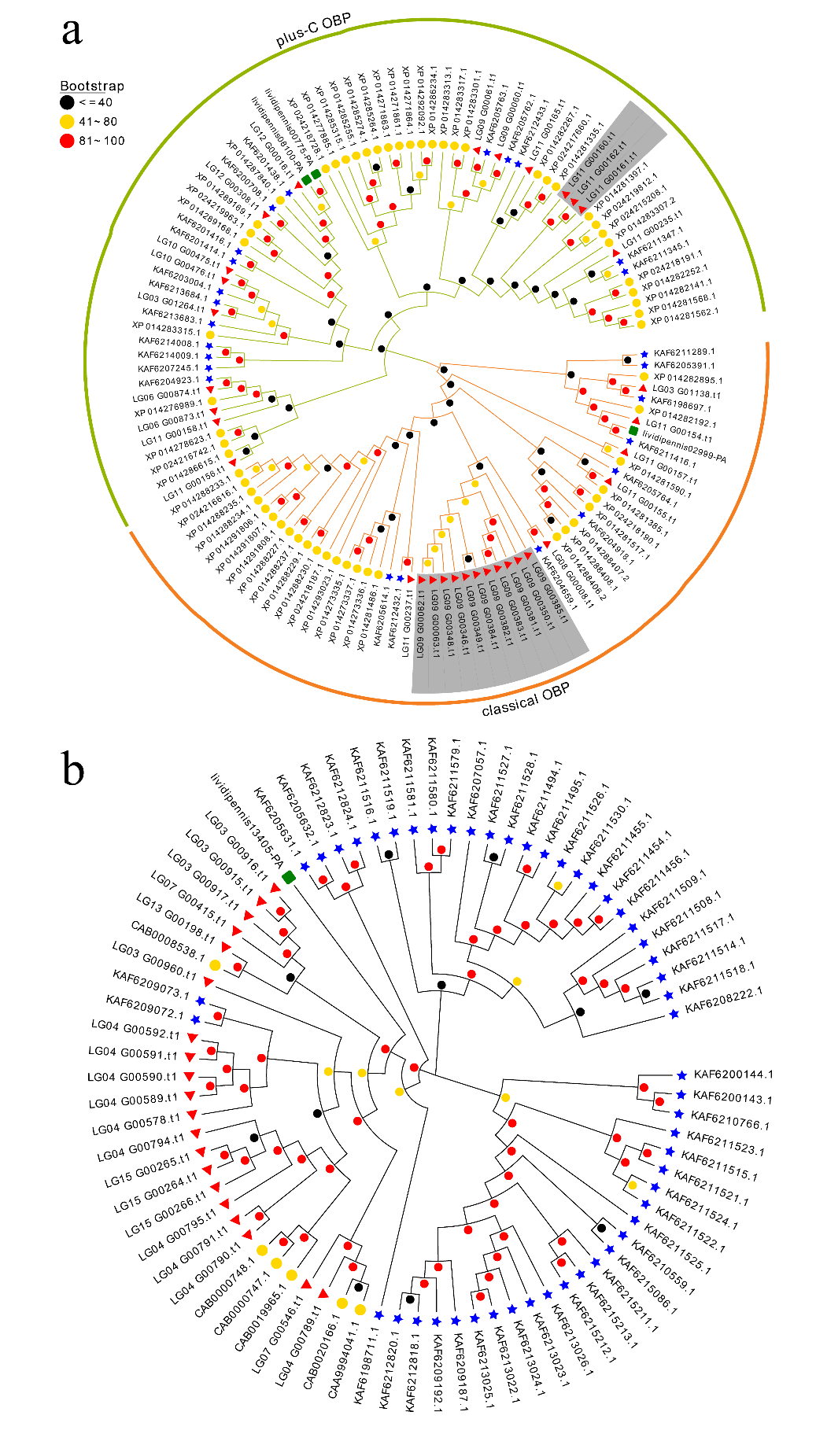
**

**Fig. S10** Phylogenetic analysis of odorant-binding proteins (OBPs) and polygalacturonases (PGs) among *Pachypeltis micranthus*, *Apolygus lucorum*, *Cyrtorhinus lividipennis*, and *Halyomorpha halys*. (a) OBPs, (b) PGs. Species-specific expanded clades in *P. micranthus* are marked with grey shading.
