# Supplementary material for: High-quality chromosome-level scaffolds of the plant bug Pachypeltis micranthus provide insights into the availability of Mikania micrantha control
Source: BMC Genomics. 2023 Jun 20;24:339. doi: 10.1186/s12864-023-09445-8 (PMC10280852; doi:10.1186/s12864-023-09445-8)
Supplement: Supplementary file 2 — Additional file 2: Table S1. Statistical information of 17-kmer analysis for the Pachypeltis micranthus genome. Table S2. Statistics of the genome assembly of Pachypeltis micranthus. Table S3. BUSCO scores of the assembled Pachypeltis micranthus genome. Table S4. CEGMA assessment results. Table S5. Statistics of the mapping rates on the genome assembly for Illumina reads. Table S6. Statistic of the accuracy of single-base in the assembled genome. Table S7. Statistics of the mapping rates on the genome assembly for Hi-C sequencing data. Table S8. Statistics of the valid paired-end reads of unique mapped paired-end reads. Table S9. Statistics of chromosome-level scaffolds in Pachypeltis micranthus genome assembly. Table S10. Statistics of repeat sequences identified in Pachypeltis micranthus genome. Table S11. Summary of protein-coding genes annotation of the genome assembly. Table S12. Comparative statistics of protein-coding genes between Pachypeltis micranthus and other Hemiptera species. Table S13. Statistics for functionally annotated protein-coding genes. Table S14. BUSCO assessment of the protein-coding genes. Table S15. Summary statistics for non-coding RNAs. Table S16. Conserved orthologs of ten species. Table S17. Statistics of species-specific genes in Pachypeltis micranthus genome. Table S18. KEGG enrichment analyses of the expanded gene families in the Pachypeltis micranthus genome. Table S19. GO enrichment analyses of the expanded gene families in the Pachypeltis micranthus genome. Table S20. GO enrichment analyses of the significantly contracted gene families in the Pachypeltis micranthus genome. Table S21. KEGG enrichment analyses of the significantly contracted gene families in Pachypeltis micranthus genome. Table S22. KEGG enrichment analysis of positively selected genes in Pachypeltis micranthus. Table S23. GO enrichment analysis of positively selected genes in Pachypeltis micranthus. Table S24. The software, versions, and parameters used for genome [file 12864_2023_9445_MOESM2_ESM.docx]

**Supplementary Materials**

**High-quality chromosome-level scaffolds of the plant bug *Pachypeltis micranthus* provide insights into the availability of *Mikania micrantha* control**

Xiafei Wang^1^, Ning Zhao^1^, Liqiong Cai^2^, Naiyong Liu^1^, Jiaying Zhu^1^, Bin Yang^1*^

^1^Key Laboratory of Forest Disaster Warning and Control of Yunnan Province, Southwest Forestry University, Kunming, China

^2^Key Laboratory for Forest Resources Conservation and Utilization in the Southwest Mountains of China, Ministry of Education, Southwest Forestry University, Kunming, China

*** Correspondence:** [yangbin48053@163.com](mailto:yangbin48053@163.com)

**Supplemental Tables**

**Table S1** Statistical information of 17-kmer analysis for the *Pachypeltis micranthus* genome.

| **K-mer** | **K-mer num** | **K-mer depth** | **Genome size (bp)** | **Heterozygous ratio (%)** |
| --- | --- | --- | --- | --- |
| 17 | 23,374,585,736 | 33 | 708,320,779 | 0.9% |

**Table S2** Statistics of the genome assembly of *Pachypeltis micranthus*.

| **Stat type** | **Preliminary assembly** | | **Polish genome** | |
| --- | --- | --- | --- | --- |
|  | **Contig length (bp)** | **Contig number** | **Contig length (bp)** | **Contig number** |
| N50 | 16,824,256 | 13 | 16,844,724 | 13 |
| N60 | 14,302,415 | 18 | 14,313,869 | 18 |
| N70 | 11,354,696 | 23 | 11,290,662 | 23 |
| N80 | 7,840,638 | 30 | 7,848,118 | 30 |
| N90 | 4,850,732 | 41 | 4,872,206 | 41 |
| Longest | 53,688,973 | 1 | 53,746,822 | 1 |
| Total | 710,812,697 | 91 | 712,715,612 | 91 |
| Length>=1kb | 710,812,697 | 91 | 712,715,612 | 91 |
| Length>=2kb | 710,812,697 | 91 | 712,715,612 | 91 |
| Length>=5kb | 710,812,697 | 91 | 712,715,612 | 91 |

**Table S3** BUSCO scores of the assembled *Pachypeltis micranthus* genome.

| **BUSCO benchmark** | **Number** | **Per cent (%)** |
| --- | --- | --- |
| Complete BUSCOs (C) | 1310 | 95.83 |
| Complete and single-copy BUSCOs (S) | 1295 | 94.73 |
| Complete and duplicated BUSCOs (D) | 15 | 1.10 |
| Fragmented BUSCOs (F) | 14 | 1.02 |
| Missing BUSCOs (M) | 43 | 3.15 |
| Total BUSCO groups searched | 1367 | 100.00 |

**Table S4** CEGMA assessment results.

| **Type** | **Complete** | | **Complete + Partial** | |
| --- | --- | --- | --- | --- |
|  | **Prots** | **Completeness (%)** | **Prots** | **Completeness (%)** |
| Total | 145 | 58.47 | 226 | 91.13 |
| Group1 | 35 | 53.03 | 56 | 84.85 |
| Group2 | 34 | 60.71 | 50 | 89.29 |
| Group3 | 33 | 54.1 | 58 | 95.08 |
| Group4 | 43 | 66.15 | 62 | 95.38 |

**Table S5** Statistics of the mapping rates on the genome assembly for Illumina reads.

| **Sample** | **Coverage Depth (X)** | **Coverage Rate (%)** | **Mapping Rate (%)** |
| --- | --- | --- | --- |
| *P. micranthus* | 154.80 | 99.70 | 97.79% |

**Table S6** Statistic of the accuracy of single-base in the assembled genome.

| **Depth (X)** | **Hetero SNP** | **Hetero Indel** | **Homo SNP** | **Error Rate by Homo SNP (%)** | **Homo Indel** | **Error Rate by Homo Indel (%)** | **Error Rate by Homo Variants (%)** | **Accuracy of Genome (%)** |
| --- | --- | --- | --- | --- | --- | --- | --- | --- |
| depth>=1x | 5,492,681 | 111,840 | 9224 | 0.001294 | 9316 | 0.001307 | 0.002601 | 99.997399 |
| depth>=5x | 5,491,879 | 110,496 | 6193 | 0.000869 | 4832 | 0.000678 | 0.001547 | 99.998453 |
| depth>=10x | 5,488,535 | 104,459 | 4016 | 0.000563 | 2348 | 0.000329 | 0.000893 | 99.999107 |

**Table S7** Statistics of the mapping rates on the genome assembly for Hi-C sequencing data.

| **Data** | **Number** |
| --- | --- |
| Clean paired-end reads | 372,400,619 |
| Unmapped paired-end reads | 73,202,648 |
| Unmapped paired-end reads rate (%) | 19.66 |
| Paired-end reads with singleton | 151,574,606 |
| Paired-end reads with singleton rate (%) | 40.70 |
| Multi-mapped paired-end reads | 27,334,737 |
| Multi-mapped ratio (%) | 7.34 |
| Unique mapped paired-end reads | 120,288,628 |
| Unique mapped ratio (%) | 32.30 |

**Table S8** Statistics of the valid paired-end reads of unique mapped paired-end reads.

| **Data** | **Number** |
| --- | --- |
| Unique mapped paired-end reads | 120,288,628 |
| Dangling end paired-end reads | 56,133,218 |
| Self-circle paired-end reads | 871,707 |
| Dumped paired-end reads | 14,833,481 |
| Valid paired-end reads | 42,362,217 |
| Valid reads of unique mapping reads (%) | 35.22 |
| Valid reads of clean reads (%) | 11.38 |

**Table S9** Statistics of chromosome-level scaffolds in *Pachypeltis micranthus* genome assembly.

| **Chromosome-level Scaffold** | **Length of Contig (bp)** | **Contig Number** |
| --- | --- | --- |
| LG01 | 85,620,765 | 6 |
| LG02 | 81,563,437 | 5 |
| LG03 | 74,867,850 | 8 |
| LG04 | 61,639,866 | 4 |
| LG05 | 55,436,040 | 3 |
| LG06 | 53,746,822 | 1 |
| LG07 | 51,037,615 | 5 |
| LG08 | 48,146,867 | 4 |
| LG09 | 33,186,119 | 6 |
| LG10 | 32,806,651 | 4 |
| LG11 | 32,427,311 | 6 |
| LG12 | 27,742,210 | 5 |
| LG13 | 24,058,039 | 7 |
| LG14 | 23,296,638 | 4 |
| LG15 | 21,931,162 | 3 |
| Total | 707,507,392 | 71 |

**Table S10** Statistics of repeat sequences identified in *Pachypeltis micranthus* genome.

| **Class** | **Order** | **Super Family** | **Number of Elements** | **Length of Sequence (bp)** | **Percentage of Sequence (%)** |
| --- | --- | --- | --- | --- | --- |
|  |  |  |  |  |  |
| Class I |  |  | 915,212 | 204,365,101 | 28.67 |
|  | LINE |  | 514,043 | 129,424,898 | 18.16 |
|  |  | R1 | 127,041 | 42,961,684 | 6.03 |
|  |  | L1 | 6891 | 988,852 | 0.14 |
|  |  | Unknown | 136,908 | 24,854,755 | 3.49 |
|  |  | CR1 | 15,557 | 3,361,962 | 0.47 |
|  |  | R2 | 33,376 | 5,802,051 | 0.81 |
|  |  | RTE-BovB | 5754 | 1,734,063 | 0.24 |
|  |  | Penelope | 31,889 | 6,731,222 | 0.94 |
|  |  | R1-LOA | 28,139 | 7,176,258 | 1.01 |
|  |  | L2 | 29,000 | 7,565,028 | 1.06 |
|  |  | RTE-X | 2961 | 1,092,771 | 0.15 |
|  |  | LOA | 68,913 | 20,621,398 | 2.89 |
|  |  | Jockey | 14,287 | 4,274,748 | 0.60 |
|  |  | I-Jockey | 6804 | 1,519,382 | 0.21 |
|  |  | Other | 6523 | 740,724 | 0.10 |
|  | LTR |  | 374,006 | 71,812,904 | 10.08 |
|  |  | Gypsy-Cigr | 4241 | 1,180,213 | 0.17 |
|  |  | Gypsy | 59,536 | 17,451,079 | 2.45 |
|  |  | Copia | 12,036 | 3,634,205 | 0.51 |
|  |  | Unknown | 256,559 | 36,915,600 | 5.18 |
|  |  | Pao | 33,751 | 12,359,152 | 1.73 |
|  |  | Other | 7883 | 272,655 | 0.04 |
|  | SINE |  | 27,163 | 3,127,299 | 0.44 |
|  |  | Unknown | 26,808 | 3,114,853 | 0.44 |
|  |  | Other | 355 | 12,446 | 0 |
| Class II |  |  | 644,220 | 140,539,782 | 19.72 |
|  | DNA |  | 630,108 | 136,804,681 | 19.19 |
|  |  | Unknown | 564,904 | 124,163,506 | 17.42 |
|  |  | Maverick | 7953 | 1,800,888 | 0.25 |
|  |  | hAT-Charlie | 4242 | 967,888 | 0.14 |
|  |  | TcMar-Tc1 | 6178 | 1,721,353 | 0.24 |
|  |  | TcMar-Mariner | 8482 | 2,220,953 | 0.31 |
|  |  | PiggyBac | 2614 | 885,858 | 0.12 |
|  |  | Other | 35,735 | 5,044,235 | 0.71 |
|  | RC |  | 6937 | 1,213,271 | 0.17 |
|  |  | Helitron | 6937 | 1,213,271 | 0.17 |
|  | MITE |  | 7175 | 2,521,830 | 0.35 |
|  |  | Unknown | 7175 | 2,521,830 | 0.35 |
| Total TEs |  |  | 1,559,432 | 344,904,883 | 48.39 |
| Tandem Repeats |  |  | 31,746 | 1,584,254 | 0.22 |
|  | tandem_repeat |  | 10,963 | 1,341,628 | 0.19 |
|  | SSR |  | 20,783 | 242,626 | 0.03 |
| Unknown |  |  | 222,575 | 28,475,004 | 4 |
| Simple repeats |  |  | 1437 | 263,321 | 0.04 |
| Other |  |  | 4369 | 566,282 | 0.08 |
| Low complexity |  |  | 96 | 24,007 | 0 |
| Total Repeats |  |  | 1,819,655 | 375,817,751 | 52.73 |

LINE: long interspersed nuclear elements; LTR: the long terminal repeat retrotransposon; SINE: short interspersed nuclear elements; TE: Transposon Elements; RC: Rolling-circle eukaryotic transposons; MITE: miniature inverted-repeat transposable element; SSR: simple sequence repeat.

**Table S11** Summary of protein-coding genes annotation of the genome assembly.

| **Gene set** | **Total Number of Genes** | **Average Gene Length(bp)** | **Average CDS Length (bp)** | **Average Exons Number per Gene** | **Average Exon Length (bp)** | **Average Intron Length (bp)** |
| --- | --- | --- | --- | --- | --- | --- |
| De novo | 9656 | 36,470.20 | 1720.00 | 9.36 | 183.80 | 4157.76 |
| RNA-seq | 7628 | 37,002.69 | 1671.64 | 9.03 | 185.21 | 4402.39 |
| Homology | 10,854 | 33,576.26 | 1403.37 | 6.56 | 213.83 | 5783.35 |
| Final set | 11,746 | 32,170.81 | 1516.18 | 7.82 | 193.96 | 4496.87 |

**Table S12** Comparative statistics of protein-coding genes between *Pachypeltis micranthus* and other Hemiptera species.

| **Species** | **Total Number of Genes** | **Average Transcript Length (bp)** | **Average CDS Length (bp)** | **Average Exons Number per Gene** | **Average Exon Length (bp)** | **Average Intron Length (bp)** |
| --- | --- | --- | --- | --- | --- | --- |
| *Pachypeltis micranthus* | 11,746 | 32,170.81 | 1516.18 | 7.82 | 193.96 | 4496.87 |
| *Nesidiocoris tenuis* | 24,668 | 7149.11 | 913.15 | 3.94 | 231.72 | 2120.54 |
| *Apolygus lucorum* | 20,111 | 22,559.88 | 1348.05 | 6.6 | 204.18 | 3786.20 |
| *Aphis gossypii* | 12,815 | 9298.47 | 1484.10 | 6.99 | 212.47 | 1305.64 |
| *Nilaparvata lugens* | 19,806 | 20,178.30 | 1330.92 | 6.19 | 214.95 | 3630.25 |
| *Diaphorina citri* | 20,085 | 8057.39 | 1101.33 | 4.91 | 224.51 | 1781.12 |
| *Halyomorpha halys* | 14,454 | 23,468.69 | 1432.56 | 7.35 | 194.89 | 3470.03 |

**Table S13** Statistics for functionally annotated protein-coding genes.

| **Type** | | **Number** | **Per cent (%)** |
| --- | --- | --- | --- |
| Annotation | Swissprot | 9193 | 78.26 |
|  | KEGG | 6211 | 52.88 |
|  | KOG | 7800 | 66.41 |
|  | GO | 7251 | 61.73 |
|  | NR | 10,454 | 89 |
| Total | Annotated | 10,836 | 92.25 |
|  | Gene | 11,746 | - |

**Table S14** BUSCO assessment of the protein-coding genes.

| **Type** | **Number** | **Per cent (%)** |
| --- | --- | --- |
| Complete BUSCOs (C) | 1315 | 96.20 |
| Complete and single-copy BUSCOs (S) | 1300 | 95.10 |
| Complete and duplicated BUSCOs (D) | 15 | 1.10 |
| Fragmented BUSCOs (F) | 10 | 0.73 |
| Missing BUSCOs (M) | 42 | 3.07 |
| Total BUSCO groups searched | 1367 | 100 |

**Table S15** Summary statistics for non-coding RNAs.

| **Type** | | **Copy Number** | **Average Length(bp)** | **Total Length(bp)** | **Percentage of Genome (%)** |
| --- | --- | --- | --- | --- | --- |
| rRNA | 18S | 8 | 2,025.50 | 16,204 | 0.0023 |
|  | 28S | 7 | 3,997.43 | 27,982 | 0.0039 |
|  | 5.8S | 10 | 155.90 | 1559 | 0.0002 |
|  | 5S | 238 | 112.39 | 26,750 | 0.0038 |
| Small RNA | snRNA | 63 | 99.10 | 6243 | 0.0009 |
|  | miRNA | 39 | 78.97 | 3080 | 0.0004 |
|  | splicing | 82 | 142.57 | 11,691 | 0.0016 |
|  | other | 13 | 249.31 | 3241 | 0.0005 |
| Regulatory | cis-regulatory elements | 40 | 48.83 | 1953 | 0.0003 |
| tRNA | | 2501 | 74.91 | 187,354 | 0.0263 |

**Table S16** Conserved orthologs of ten species.

| **Species** | **Single-copy orthologs** | **Multiple-copy**  **orthologs** | **Unique paralogs** | **Other orthologs** | **Unclustered genes** |
| --- | --- | --- | --- | --- | --- |
| *Pachypeltis micranthus* | 896 | 2487 | 293 | 7140 | 930 |
| *Aphis glycines* | 896 | 2687 | 1634 | 7942 | 5199 |
| *Aphis gossypii* | 896 | 2648 | 145 | 7860 | 1266 |
| *Apolygus lucorum* | 896 | 2936 | 2513 | 9493 | 4273 |
| *Cimex lectularius* | 896 | 2418 | 463 | 6565 | 1594 |
| *Diaphorina citri* | 896 | 3026 | 2466 | 6277 | 7420 |
| *Halyomorpha halys* | 896 | 2726 | 1170 | 7300 | 2362 |
| *Nilaparvata lugens* | 896 | 3278 | 2395 | 8382 | 4855 |
| *Nesidiocoris tenuis* | 896 | 3264 | 6000 | 5474 | 9034 |
| *Rhopalosiphum maidis* | 896 | 2633 | 261 | 7673 | 597 |

**Table S17** Statistics of species-specific genes in *Pachypeltis micranthus* genome.

| **Species** | **Unique Gene** | **Unclustered** | **Species-specific Genes** |
| --- | --- | --- | --- |
| *Pachypeltis micranthus* | 293 | 930 | 1223 |

**Table S18** KEGG enrichment analyses of the expanded gene families in the *Pachypeltis micranthus* genome.

| **Map Number** | | **Pathway** | **Count** | ***P-value*** | ***Q-value*** |
| --- | --- | --- | --- | --- | --- |
| map00040 | pentose and glucuronate interconversions | | 53 | 1.28E-72 | 4.10E-71 |
| map01110 | biosynthesis of secondary metabolites | | 47 | 4.87E-29 | 1.73E-28 |
| map00830 | retinol metabolism | | 34 | 8.32E-47 | 1.33E-45 |
| map00140 | steroid hormone biosynthesis | | 34 | 1.74E-45 | 1.86E-44 |
| map00053 | ascorbate and aldarate metabolism | | 34 | 1.13E-44 | 9.04E-44 |
| map00860 | porphyrin and chlorophyll metabolism | | 34 | 1.70E-42 | 1.09E-41 |
| map00982 | drug metabolism-cytochrome p450 | | 34 | 1.85E-38 | 9.84E-38 |
| map00980 | metabolism of xenobiotics by cytochrome p450 | | 34 | 3.26E-38 | 1.49E-37 |
| map00983 | drug metabolism-other enzymes | | 34 | 9.17E-35 | 3.67E-34 |
| map04340 | hedgehog signalling pathway | | 8 | 5.20E-07 | 1.39E-06 |
| map04341 | hedgehog signalling pathway-fly | | 8 | 1.34E-06 | 3.30E-06 |
| map00561 | glycerolipid metabolism | | 8 | 2.28E-05 | 5.21E-05 |
| map01220 | degradation of aromatic compounds | | 7 | 8.65E-10 | 2.77E-09 |
| map00930 | caprolactam degradation | | 7 | 4.33E-09 | 1.26E-08 |
| map00010 | glycolysis/gluconeogenesis | | 7 | 0.000233 | 0.000466 |
| map00900 | terpenoid backbone biosynthesis | | 6 | 6.07E-05 | 0.000129 |
| map04210 | apoptosis | | 6 | 0.009447 | 0.016795 |
| map04612 | antigen processing and presentation | | 5 | 0.003903 | 0.007347 |
| map04145 | phagosome | | 5 | 0.042538 | 0.064820 |
| map04140 | autophagy-animal | | 5 | 0.084366 | 0.107989 |
| map04611 | platelet activation | | 4 | 0.031623 | 0.053260 |
| map04080 | neuroactive ligand-receptor interaction | | 4 | 0.035683 | 0.057093 |
| map04072 | phospholipase d signalling pathway | | 4 | 0.060332 | 0.083940 |
| map04810 | regulation of actin cytoskeleton | | 4 | 0.095615 | 0.117680 |
| map00790 | folate biosynthesis | | 1 | 0.432898 | 0.506682 |
| map00051 | fructose and mannose metabolism | | 1 | 0.459181 | 0.506682 |
| map00981 | insect hormone biosynthesis | | 1 | 0.459181 | 0.506682 |
| map04620 | toll-like receptor signalling pathway | | 1 | 0.508192 | 0.536509 |
| map04380 | osteoclast differentiation | | 1 | 0.519743 | 0.536509 |
| map00052 | galactose metabolism | | 1 | 0.542047 | 0.542047 |

**Table S19** GO enrichment analyses of the expanded gene families in the *Pachypeltis micranthus* genome.

| **GO ID** | **GO term** | **GO Class** | **Count** | ***P-value*** | ***Q-value*** | |
| --- | --- | --- | --- | --- | --- | --- |
| GO:0016758 | transferase activity, transferring hexosyl groups | MF | 34 | 1.28E-72 | | 4.10E-71 |
| GO:0016020 | membrane | CC | 31 | 4.87E-29 | | 1.73E-28 |
| GO:0006508 | proteolysis | BP | 30 | 8.32E-47 | | 1.33E-45 |
| GO:0005549 | odorant binding | MF | 23 | 1.74E-45 | | 1.86E-44 |
| GO:0008234 | cysteine-type peptidase activity | MF | 20 | 1.13E-44 | | 9.04E-44 |
| GO:0022857 | transmembrane transporter activity | MF | 20 | 1.70E-42 | | 1.09E-41 |
| GO:0055085 | transmembrane transport | BP | 20 | 1.85E-38 | | 9.84E-38 |
| GO:0050909 | sensory perception of taste | BP | 18 | 3.26E-38 | | 1.49E-37 |
| GO:0006030 | chitin metabolic process | BP | 13 | 9.17E-35 | | 3.67E-34 |
| GO:0008061 | chitin binding | MF | 13 | 5.20E-07 | | 1.39E-06 |
| GO:0005576 | extracellular region | CC | 13 | 1.34E-06 | | 3.30E-06 |
| GO:0004650 | polygalacturonase activity | MF | 11 | 2.28E-05 | | 5.21E-05 |
| GO:0004984 | olfactory receptor activity | MF | 11 | 8.65E-10 | | 2.77E-09 |
| GO:0007608 | sensory perception of smell | BP | 11 | 4.33E-09 | | 1.26E-08 |
| GO:0005975 | carbohydrate metabolic process | BP | 11 | 0.000233 | | 0.000466 |
| GO:0004252 | serine-type endopeptidase activity | MF | 10 | 6.07E-05 | | 0.000129 |
| GO:0000166 | nucleotide binding | MF | 8 | 0.009447 | | 0.016794 |
| GO:0003887 | DNA-directed DNA polymerase activity | MF | 7 | 0.003902 | | 0.007346 |
| GO:0006260 | DNA replication | BP | 7 | 1.28E-72 | | 4.10E-71 |
| GO:0008299 | isoprenoid biosynthetic process | BP | 6 | 4.87E-29 | | 1.73E-28 |
| GO:0046983 | protein dimerization activity | MF | 5 | 0.091589 | | 0.117757 |
| GO:0008289 | lipid binding | MF | 1 | 0.382356 | | 0.448852 |
| GO:0016787 | hydrolase activity | MF | 1 | 0.956456 | | 0.956456 |

**Table S20** GO enrichment analyses of the significantly contracted gene families in the *Pachypeltis micranthus* genome.

| **GO ID** | **GO term** | **GO class** | **Count** | ***P-value*** | ***Q-value*** |
| --- | --- | --- | --- | --- | --- |
| GO:0006352 | DNA-templated transcription | BP | 1 | 0.002620 | 0.005240 |
| GO:0005634 | nucleus | CC | 1 | 0.028961 | 0.028961 |

**Table S21** KEGG enrichment analyses of the significantly contracted gene families in the *Pachypeltis micranthus* genome.

| **Map number** | **Pathway** | **Count** | ***P-value*** | ***Q-value*** |
| --- | --- | --- | --- | --- |
| map03022 | basal transcription factors | 1 | 0.010743 | 0.010743 |

**Table S22** KEGG enrichment analysis of positively selected genes in *Pachypeltis micranthus*.

| **Map Number** | **Pathway** | | **Count** | ***P-value*** | ***Q-value*** |
| --- | --- | --- | --- | --- | --- |
| map00062 | fatty acid elongation | 1 | | 0.030962 | 0.108691 |
| map01040 | biosynthesis of unsaturated fatty acids | 1 | | 0.033870 | 0.108691 |
| map03440 | homologous recombination | 1 | | 0.038220 | 0.108690 |
| map03460 | fanconi anaemia pathway | 1 | | 0.048307 | 0.108691 |
| map01212 | fatty acid metabolism | 1 | | 0.076663 | 0.125468 |
| map03018 | RNA degradation | 1 | | 0.083645 | 0.125468 |
| map04910 | insulin signalling pathway | 1 | | 0.134012 | 0.172301 |
| map04142 | lysosome | 1 | | 0.164187 | 0.184710 |
| map01110 | biosynthesis of secondary metabolites | 1 | | 0.401731 | 0.401731 |

**Table S23** GO enrichment analysis of positively selected genes in *Pachypeltis micranthus*.

| **GO ID** | **GO term** | **GO Class** | **Count** | ***P-value*** | | ***Q-value*** |
| --- | --- | --- | --- | --- | --- | --- |
| GO:0003677 | DNA binding | MF | 2 | 0.069880 | 0.139760 | |
| GO:0016627 | oxidoreductase activity, acting on the CH-CH group of donors | MF | 1 | 0.023350 | 0.139760 | |
| GO:0006396 | RNA processing | BP | 1 | 0.040244 | 0.139760 | |
| GO:0008168 | methyltransferase activity | MF | 1 | 0.054517 | 0.139760 | |
| GO:0006629 | lipid metabolic process | BP | 1 | 0.069766 | 0.139760 | |
| GO:0004930 | G-protein coupled receptor activity | MF | 1 | 0.130797 | 0.180089 | |
| GO:0007186 | G-protein coupled receptor signalling pathway | BP | 1 | 0.140614 | 0.180089 | |
| GO:0003723 | RNA binding | MF | 1 | 0.159955 | 0.180089 | |
| GO:0043565 | sequence-specific DNA binding | MF | 1 | 0.162080 | 0.180089 | |
| GO:0006355 | regulation of transcription, DNA-templated | BP | 1 | 0.297658 | 0.297657 | |

**Table S24** The software, versions, and parameters used for genome assembly and annotation.

| **Software** | **Version** | **Parameter** |
| --- | --- | --- |
| **Assembly** |  |  |
| NextDenovo | v2.3.1 | reads_cutoff:1k, seed_cutoff:29k |
| NextPolish | v1.3.0 | default |
| BUSCO | 4.0.5 | -l insecta_odb10 -g genome |
| CEGMA | v2 | default |
| BWA | v0.7.12 | default |
| samtools | v1.4 | defaut |
| BCFtools | v1.8.0 | default |
| Minimap2 | v2.24 | -x map-ont |
| Hisat2 | v2.1.0 | default |
| fastp | v0.20.0 | default |
| Bowtie2 | v2.3.2 | -end-to-end --very-senstive -L 30 |
| Hi-C-Pro | v2.8.1 | default |
| LACHESIS | - | default |
| MCScanX | - | default |
| **Annotation** |  |  |
| RepeatModeler | v1.0.11 | -engine wublast |
| LTR_FINDER | - | default |
| MITE-Hunter | - | -n 20 -P 0.2 -c 3 |
| RepeatMasker | v1.331 | nolow -no_is -gff -norna -engine abblast -lib lib |
| GeMoMa | v1.6.1 | default |
| STAR | v2.7.3 | default |
| Augustus | v3.3.1 | default |
| EVidenceModeler | v1.1.1 | --segmentSize 1000000 --overlapSize 100000 |
| TransposonPSI | - | default |
| PASA | v2.3.3 | -c alignAssembly.config -C -R -g genome.fasta -T -u trans.fasta -t trans.clean.fasta -f fl.acc --CPU 10 --ALIGNERS gmap |
| tRNAscan-SE | v2.0 | --thread 4 -E -I |
| RNAmmer | v1.2 | -S euk -m lsu,ssu,tsu -gff |
| Infernal | v1.1.2 | default |

**Table S25** Calibrating time for estimating divergence times (Million years ago, Mya).

| **Clade** | **Clade** | **Min (Mya)** | **Max (Mya)** |
| --- | --- | --- | --- |
| *Aphis glycines* | *Aphis gossypii* | 1.4 [1] | 13.3 [1] |
| *Aphis gossypii* | *Rhopalosiphum maidis* | 30.1 [1] | 52.6 [2] |
| *Diaphorina citri* | *Nilaparvata lugens* | 112.5 [3] | 391.7 [4] |
| *Nilaparvata lugens* | *Halyomorpha halys* | 234.9 [5] | 366.2 [6] |

**References:**

1. Kim H, Zahir T, Tator CH, Shoichet MS. Effects of dibutyryl Cyclic-AMP on survival and neuronal differentiation of neural stem/progenitor cells transplanted into spinal cord injured rats. Plos One. 2011;6(6):12.

2. Hardy NB, Peterson DA, von Dohlen CD. The evolution of life cycle complexity in aphids: Ecological optimization or historical constraint? Evolution. 2015;69(6):1423-1432.

3. Smith VS, Ford T, Johnson KP, Johnson PCD, Yoshizawa K, Light JE. Multiple lineages of lice pass through the K-Pg boundary. Biology Letters. 2011;7(5):782-785.

4. Wheat CW, Wahlberg N. Phylogenomic insights into the cambrian explosion, the colonization of land and the evolution of flight in arthropoda. Systematic Biology. 2013;62(1):93-109.

5. Song N, Liang A. A preliminary molecular phylogeny of planthoppers (Hemiptera: Fulgoroidea) based on nuclear and mitochondrial DNA sequences. Plos One. 2013;8(3):11.

6. Rainford JL, Hofreiter M, Nicholson DB, Mayhew PJ. Phylogenetic distribution of extant richness suggests metamorphosis is a key innovation driving diversification in insects. Plos One. 2014;9(10):7.
